# Do small hospitals have lower quality? Evidence from the English NHS

**DOI:** 10.1016/j.socscimed.2020.113500

**Published:** 2020-11

**Authors:** James Gaughan, Luigi Siciliani, Hugh Gravelle, Giuseppe Moscelli

**Affiliations:** a Department of Economics and Related Studies, University of York, York, YO10 5DD, UK; b Centre for Health Economics, University of York, York, YO10 5DD, UK; c Department of Economics, University of Surrey, Guildford, Surrey, GU2 7XH, UK

**Keywords:** Quality, Small hospitals, National health service, England

## Abstract

We investigate the extent to which small hospitals are associated with lower quality. We first take a patient perspective, and test if, controlling for casemix, patients admitted to small hospitals receive lower quality than those admitted to larger hospitals. We then investigate if differences in quality between large and small hospitals can be explained by hospital characteristics such as hospital type and staffing. We use a range of quality measures including hospital mortality rates (overall and for specific conditions), hospital acquired infection rates, waiting times for emergency patients, and patient perceptions of the care they receive. We find that small hospitals, with fewer than 400 beds, are generally not associated with lower quality before or after controlling for hospital characteristics. The only exception is heart attack mortality, which is generally higher in small hospitals.

## Highlights

• We investigate whether small hospitals are associated with lower quality indicators.
• We use patient mortality, infections, A&E waiting times, care perception as quality.
• Small hospitals (<400 beds) are not generally associated with lower quality.
• However, AMI mortality is generally higher in small hospitals.
• Our findings do not support closures of small hospitals to achieve quality gains.

## 1 Introduction

Quality of care is a key policy objective in health systems across OECD countries. It is multifaceted: it includes clinical quality, patient experience (such as being treated with respect and being able to communicate and have a dialogue with the doctor), amenities, and health system responsiveness and availability of services (such as how long patients need to wait for health care) (Busse et al., 2019; De Pouvourville and Minvielle, 2003).

*Small* hospitals, defined in the context of the English National Health Service (NHS) as those with under 400 beds, are regularly the subject of debate and policy intervention (Vaughan et al., 2018; Imison, 2018). One policy concern is that small hospitals are not able to exploit scale and scope economies to be financially sustainable (Monitor, 2014) and may provide lower quality of care, leading to higher mortality (overall and for specific conditions), higher hospital acquired infection rates, and worse patient perceptions of their care. This may be reinforced by greater difficulty for small hospitals in recruiting and attracting staff, managing a smaller pool of staff that guarantees 24/7 provision of emergency care, or investing in diagnostic services (Vaughan et al., 2018). Small hospitals can also be targeted when cost-containment measures are introduced, and are often the subject of hospital service reconfigurations leading to closure or mergers (Monitor, 2014; Edwards, 2016; Vaughan et al., 2018). Small hospitals tend to be located in less urbanised areas, and their closure has a potential impact on the accessibility of services (Monitor, 2014).

Low quality of services by small hospitals is a key argument to justify the closure of a small hospital, despite the accessibility costs that this implies (Vaughan et al., 2018). Proximity to hospitals is a key driver of hospital choice (Gaynor et al., 2016; Gutacker et al., 2016). Quality in small hospitals therefore also raises equity concerns if patients living close to them systematically receive lower-quality care relative to patients using larger providers (Scobie and Morris, 2020). Closure of small hospitals will also have equity implications if patients have to travel long distances (Buchmueller et al., 2006) and have poorer access to care.

We investigate whether small hospitals are associated with lower quality in England, thereby informing policy on hospital closures and reconfigurations. Hospital configuration is already concentrated in England, with about 140 acute hospital organizations, covering a population of 56 million. We define hospitals as small if they have less than 400 beds; a group accounting for 8% of hospitals. Very large hospitals with more than 1150 beds are about 17% of hospitals.

We use twelve quality measures covering clinical quality, patient reported outcomes, and waiting times as a measure of responsiveness to patients. We include five hospital mortality rates (overall, non-elective and for three specific conditions: AMI, hip fracture and stroke), two measures of hospital-acquired infection rates (MRSA and C-Difficile), four patient reported perceptions of the care they receive (hospital cleanliness, decision involvement, being treated with dignity, recommendable to friends and family), and waiting times in the accidents and emergency department.

Since patients are concerned if they receive low quality of care, regardless of its cause, we first investigate whether, for a given casemix, patients treated in small hospitals receive lower quality than those in larger hospitals.

Second, we investigate if any association of quality with hospital size can be explained by hospital characteristics. It is important from a policy perspective to understand whether, if lower quality is observed in small hospitals, this is due to the size of the hospital or to other factors associated with hospital size and which could be influenced directly by policy. These other factors include hospital characteristics (e.g. being a teaching hospital, or having Foundation Trust status, which gives more independence to the hospital in managing resources), staff composition, input costs, market structure, the aggregate demand faced by the hospital (as affected by the proportion of elderly and income deprivation in the hospital catchment area), and the accessibility of primary care which can be a substitute for secondary care.

### 1.1 Related literature

The voluminous literature on the volume-outcome relationship examines the hypothesis that doctors or hospitals with higher volumes for a specific treatment have better health outcomes because of learning-by-doing effects or scale economies. Depending on the treatment, the empirical findings are mixed or provide only weak support for a positive effect of size. See for example recent systematic reviews on hip fracture (Wiegers et al., 2019); colorectal cancer (Huo et al., 2017); carotid endarterectomy (Phillips et al., 2018). Our study differs from this literature because it takes a hospital-level perspective. We investigate whether hospitals with low overall size provide lower quality generally, not whether hospitals with small volumes for specific treatments have worse quality for those treatments.

Our study also relates to the literature on hospital mergers. Small hospitals can be targeted in mergers to achieve synergies and scale economies. For example, in France 90 mergers have been approved since 1995, mainly involving small or medium-sized hospitals (Siciliani et al., 2017). The evidence does not suggest that mergers improve quality. In England, 112 hospitals merged between 1997 and 2006. Gaynor et al. (2012) found that these mergers did not affect clinical quality and productivity, but did reduce activity and increased waiting times. The evidence from the US also suggests that mergers do not improve quality (Mutter et al., 2011).

A study by a health regulator in England (Monitor, 2014) focussed on the relationship between size (having over 700 beds) and financial performance, but also included mortality and patient experience. Univariate regression models were estimated for 69 single site hospitals in 2012/13 and found no consistent relationship between size and quality. We use multiple regression analyses of up to five years of data on up to 148 hospital trusts, with a larger set of quality measures and explanatory factors in addition to size.

### 1.2 Institutional background

NHS hospital treatment is tax funded and there are no patient charges. Patients require a referral from their general practitioner (GP) to access elective hospital care. Most hospital care for NHS patients is provided by public hospitals (NHS Trusts) covering both emergency and elective care. These are public bodies subject to tight financial and regulatory control and are the only providers of emergency care. The organizational structure of NHS Trusts varies because they can have one or more sites, be teaching or non-teaching, and may be Foundation Trusts which have looser financial and other constraints.

Prospective payment per patient was rolled out between 2003 and 2009. It is based on Healthcare Resource Groups (HRGs), which are the English analogue of Diagnosis Related Groups (DRGs). These are groups of hospital care services sufficiently homogeneous to receive the same reimbursement (Mason et al., 2011). A tariff for each HRG is calculated from the national average cost of treating the same patient group three years before, adjusted for inflation. In addition, a hospital-specific Market Forces Factor adjusts for unavoidable cost differences due to the hospital's location (Monitor, 2013; Grašič et al., 2015). Hospitals can increase profit (or reduce deficits) if they attract more patients with a tariff greater than their marginal cost.

## 2 Data

We use publicly-available data at the hospital organization (i.e. *hospital Trust*) level for five financial years (1st April to 31st March) from 2010/11 to 2014/15: 144 hospitals in 2010–11, 145 in 2011–12, 141 in 2012–13, 140 in 2013–14, and 134 in 2014–15. Detailed variable definitions are in the online Appendix A.1.

### 2.1 Dependent variables

We use five measures of mortality, two measures of hospital acquired infection, four patient reported measures of perceptions of the care, Accident and Emergency waiting times, and a measure of hospital costs.

The five measures of risk-adjusted mortality are hospital mortality as captured by the Summary Hospital Mortality Indicator (SHMI) for all patients (emergency and elective); the mortality rate of patients receiving a broad set of non-elective procedures (Appendix A.2, and Appendix 5 of HSCIC (2016)); mortality rates after emergency admissions for three specific conditions: Acute Myocardial Infarction (AMI, more commonly known as heart attack), hip fracture and stroke. These three high-volume conditions have non-negligible mortality risk: 30-day mortality in England in 2002–2011 was 7% for AMI, 3.5% for hip fracture and 16.5% for stroke (Moscelli et al., 2018b).

The five mortality measures are for deaths in hospital or within 30 days of discharge. All are standardised by age and gender and some also by admission diagnosis and co-morbidities. (NHS Digital, 2016a, NHS Digital, 2016b). SHMI is available for all acute non-specialist hospitals and is an indirectly standardised (actual/expected) ratio with a mean of 100 in each year. The other mortality measures are indirectly standardised rates per 100 patients for acute hospitals treating patients with the relevant diagnosis. Except for SHMI, which covers both emergency and non-emergency conditions, the mortality measures are for patients admitted as emergencies. Emergency patients are usually treated in the closest hospital. By contrast, elective (non-emergency) patients in England can choose their hospital and this may result in a biased measure of quality if patients’ choice is affected by unobserved patient morbidity (Gowrisankaran and Town, 1999; Moscelli et al., 2018a).

The two hospital-acquired infections measures are (i) the number of Methicillin-Resistant Staphylococcus Aureus (MRSA) infections and (ii) Clostridium Difficile (C-Difficile) infections per 100,000 occupied bed days (Public Health England, 2016). Details are in Appendix A.1.

We use four indicators of patient perceptions of quality. Three are derived from responses to questions in the annual inpatient survey by the sector regulator (Care Quality Commission, (CQC), 2016). The questions relate to patient views on cleanliness (“In your opinion, how clean was the hospital room or ward that you were in?“), involvement in decision-making (“Were you involved as much as you wanted to be in decisions about your care and treatment?“) and dignity while in hospital (“Overall, did you feel you were treated with respect and dignity while you were in the hospital?“) (The King’s Fund, 2015). The wording of these questions did not vary over time. Values range from 0 to 100 and are standardised by age, gender, ethnicity and route of admission. These variables were available for the first four years of our sample period, 2010/11–2013/14. We also use the percentage of patients who would recommend the Trust where they were treated to family and friends, which is published by NHS England for 2014/15. A similar variable is available for 2013/14, but measured differently (see Appendix Table A1). We use the 2014/15 data in our main analysis and report the results for 2013/14 in the online Appendix D.

As a measure of patient responsiveness, we use the percentage of patients waiting more than 4 h in an A&E Department, from attendance to admission, discharge, or death. Keeping this rate below 5% is a key target for NHS Trusts and is publicised in the media.

Since it has been suggested that small hospitals are less efficient in providing healthcare, we also use hospital average costs – the Reference Cost Index (RCI) – as a dependent variable. The RCI is a measure of cost for each hospital after allowing for local input prices over which a hospital has no control and for the number of patients treated.

### 2.2 Independent variables

Our key explanatory variable is the size of hospital, measured as the number of beds. NHS England reports the average number of overnight and day-case beds in each hospital for each quarter (NHS England, 2015). We use the sum of overnight and day-case beds averaged across four quarters in each year when the quality measures are only available at the annual level. For two of the quality measures (Friends and Family test, A&E waiting times) we have quarterly data and therefore estimate models using the quarterly beds data.

To estimate a flexible relationship between size and quality we use seven bed size categories: less than 400, 400–549, 550–699, 700–849, 850–999, 1000–1149, more than 1150. The 150-beds bands are sufficiently small to flexibly allow for a non-linear relationship between size and quality, and at the same time large enough to provide a reasonable number of observations within each band, with the smallest band having over 50 hospital-year observations (7.7% of the sample).

Characteristics of hospital patients are derived from Hospital Episode Statistics (HES). We use the proportion of admissions that are emergencies, the proportion of male patients, and the proportions in seven age bands (0–15, 16–29, 30–44, 45–59, 60–74, 75–89 and 90+). These control for patient casemix for hospital acquired infections, waits in accident and emergency, friends and family test, and RCI and further control for residual casemix for the other outcome variables.

Amongst hospital characteristics, we include a (1,0) indicator for the hospital Trust having teaching status, since teaching hospitals may attract better motivated and qualified staff. We also include an indicator for Foundation Trust status, which gives hospitals more independence and discretion in managing any financial surplus. The two indicators are not mutually exclusive, with some hospitals having both Foundation and teaching status. To improve the homogeneity of our sample we do not include specialised hospitals (e.g. orthopaedic hospitals).

Staff skill mix may affect quality if, for example, greater availability of doctors improves diagnosis. We therefore control for this using the percentages of full-time equivalent (FTE) staff classified as doctors, nurses and midwives, and managers. Staff variables are the yearly means of monthly snapshots (taken on the last day of each month from the Electronic Staff Record) by NHS Digital.

We use data from the Organization Data Service to control for the number of sites in each hospital (categorised as 1, 2, 3, 4+ sites) since hospitals with more sites may be more difficult to organise and manage and so produce worse quality. We conduct the analysis at the Trust (hospital) rather than the site level. This is because we use publicly-available risk-adjusted quality indicators that are reported at the Trust, not the site, level. Moreover, some of our control variables (patient age groups, emergency admissions and staff) are publicly available only at Trust level.

We also include variables that capture features of the catchment area around the hospital. We use a catchment area of 30 km, following previous literature (Cooper et al., 2011; Gaynor et al., 2013). We attribute data to hospitals from Lower Super Output Areas (LSOAs) with centroids within 30 km of the headquarters of the hospital Trust. There are over 32,000 LSOAs in England, with an average population of 1500 and a minimum of 1000. We compute, for each hospital catchment area, the proportion of people aged 65 using mid-year Office of National Statistics (ONS) population estimates. We also attribute catchment area LSOA income deprivation from the 2015 Index of Multiple Deprivation (IMD) to hospitals. Hospitals serving older and more deprived populations may face higher demand pressures that could reduce quality.

We use the Market Forces Factor (MFF) to allow for unavoidable geographical differences in the cost of labour and capital. Higher values indicate additional unavoidable costs. We also control for market structure by including a competition measure used in other studies: the equivalent number of rivals within 30 km (Moscelli et al., 2018b; Longo et al., 2019). This is the inverse of the Herfindahl-Hirschman Index (HHI) calculated using the predicted market shares of hospitals in the hospital catchment area and is available for 2011/12 (Moscelli et al., 2018b, Appendix A).

Finally, since primary care may be a substitute for hospital care, we control for the average distance to the nearest GP for all individuals in the hospital catchment area to capture accessibility to primary care.

## 3 Methods

We employ linear regression models to investigate the relationship between quality and hospital size:

(1) $$y_{it}=\alpha +S_{it}^{\prime }\gamma_{k}+X_{it}^{\prime }\beta_{1}+H_{it}^{\prime }\beta_{2}+\mu_{t}+\mu_{i}+\varepsilon_{it},$$

where $y_{it}$ is quality in hospital iin year (or quarter) t. $S_{it}$ is our key regressor: a set of indicators for hospital bed size categories. The baseline category, to which the other six categories are compared, is 0–399 beds. The coefficients *γ*_*k*_ (*k* = 1, …,6) are the effect of size category *k* relative to the 0–399 beds category. $X_{it}$ is a vector of patient characteristics. $H_{it}$ is a vector of characteristics of the hospital and its catchment area. $\mu_{t}$ are year (or quarter) effects. $\mu_{i}$ are hospital random effects capturing unobserved hospital factors. $\varepsilon_{it}$ is an idiosyncratic error term. All models have hospital cluster robust standard errors.

We first estimate, using ordinary least squares (OLS), a special case of (1) which includes only bed categories patient characteristics $X_{it}$and year or quarter effects $\mu_{t}$. This specification, (Model 1) takes the *patient* perspective. It tests whether patients admitted to a smaller hospital have lower quality relative to patients admitted to larger hospitals, controlling for patient casemix and time period. Model 1 does not investigate why any such association arises and so does not control for hospital and catchment area characteristics which may affect quality and vary systematically with hospital size. In Model 1 the size coefficients reflect the direct association of size with quality, but they also pick up the indirect associations arising because size is correlated with hospital and catchment area factors not included in the model.

We then estimate Model 2 – the full version of (1) − which also controls for observable hospital and catchment area characteristics, and allows for random unobserved time-invariant hospital characteristics. The coefficients on the size categories now pick up the association between size and quality after allowing for potential demand and supply factors omitted from Model 1 and which may affect hospital quality.

In Model 2 we include hospital random effects to allow for unobserved time-invariant hospital characteristics. The alternative fixed effects specification would estimate the association of year-to-year *changes* in quality and *changes* in size. Because the number of beds does not vary much over time, the fixed effects specification would produce very imprecise estimates of the association. Moreover, our focus is to establish if small hospitals are associated with lower quality, not whether variations of hospital beds *over time* is associated with changes in quality within the same hospital.

We report a number of sensitivity analyses in the Online Appendix. We estimate a within-between random effects specification (Allison 2009, Schunck, 2013), which includes both the means (***S***_*i*_, ***X***_i_, ***H***_***i***_*,* ***μ*)** of the explanatory variables over time and the deviations from their means (***S***_*it*_ *−* ***S***_*i*_, ***X***_*it*_ − ***X***_*i*_, ***H***_*it*_ *−* ***H***_*i*_*,* ***μ***_***t***_ ***- μ***) of those explanatories which vary over time. The coefficients on the means reflect the relationship between quality and unobserved time-invariant hospital characteristics. As in the closely related Mundlak (1978) correlated random effects specification, the coefficients on the deviations of the time-varying terms from their means are identical to those which would be obtained by estimating a fixed effects specification which controls for unobserved time-invariant hospital characteristics correlated with size and quality. But the within-between specification also permits estimation of the effects of variables which do not change over time, such as distance to general practices and income deprivation. A fixed effects specification would drop these variables as they would be perfectly correlated with the time-invariant hospital effects.

Both Models 1 and 2 include a measure of the number of sites in a hospital. As a robustness check, we also estimate Model 2 with a restricted sample using only single-site hospitals. Finally, we also estimate a fractional logit model to allow for the fact that dependent variables are percentages, rates, or ratios and so bounded below or above.

## 4 Results

### 4.1 Descriptive statistics

Table 1 reports descriptive statistics. Mortality rates for AMI, hip fracture and stroke are 5.3%, 7.1% and 17.1% and the overall mortality rate for non-elective treatments is 3.7%. SHMI mortality is the ratio of observed to expected deaths, normalised to 100, with a standard deviation of 9.7. The mean rates of MRSA and C-Difficile hospital-acquired infections are 1.2 and 19.4 cases per 100,000 occupied bed days. 87% of patients are satisfied with the cleanliness of the hospital, 71% with involvement in decision, and 88% with being treated with dignity. The Reference Cost Index mean is 98.9 with a standard deviation of 6.2.

**Table 1:** Descriptive statistics. 2010/11 to 2014/15.

	mean	sd (overall)	sd (between)	sd (within)	min	max	N
*Dependent Variables*							
Overall mortality (SHMI)	100.00	9.72	9.13	3.95	53.92	124.70	704
AMI mortality rate	5.31	2.00	1.78	1.34	1.68	16.40	495
Non-elective mortality rate	3.68	0.71	0.59	0.41	1.86	6.45	686
Hip fracture mortality rate	7.06	1.84	1.33	1.33	2.44	14.58	665
Stroke mortality rate	17.08	3.14	2.28	2.25	6.59	32.70	678
MRSA infections/100000 bed days	1.23	1.03	0.67	0.81	0.00	5.80	702
C-Difficile infections/100000 bed days	19.45	9.30	5.42	7.68	1.60	73.60	704
Reference Cost Index (RCI)	98.89	6.22	5.36	3.26	78.01	87.94	704
Friends and family test (FFT) score^a^	70.68	8.69	8.08	3.26	22.50	91.00	560
Friends and family test (FFT) recommendation rate^a^	93.97	3.27	2.92	1.46	73.41	98.87	532
Hospital cleanliness^b^	87.39	2.76	2.47	1.26	77.25	95.78	568
Patient involvement^b^	71.33	3.22	2.53	1.99	61.80	79.85	568
Patient treated with dignity^b^	87.83	2.33	1.97	1.27	80.95	93.87	568
A&E waiting times > 4 h^a^	5.58	3.34	1.84	2.80	0.44	28.42	2235
*Patient Characteristics*							
% Patient Age 0–14	10.94	3.70	3.65	0.74	0.04	28.62	704
% Patient Age 15–29	11.87	2.37	2.40	0.78	5.25	28.19	704
% Patient Age 30–44	14.10	3.04	3.02	0.67	8.70	26.26	704
% Patient Age 45–59	15.86	1.79	1.76	0.48	10.97	23.09	704
% Patient age 60–74	22.33	3.18	3.24	0.57	10.26	29.59	704
% Patient Age 75–89	21.37	3.87	3.88	0.80	9.42	35.97	704
% Patient Age 90+	3.53	0.99	0.93	0.34	0.92	6.87	704
% Patients Male	43.42	2.65	2.62	0.74	32.51	52.45	704
% Admissions Emergencies	37.32	5.47	5.18	1.69	15.48	52.28	704
*Trust Characteristics*							
Beds in Trust (continuous)	827	378	383	60	259	2478	704
Beds in Trust <400 (“small hospital”	0.08	0.27	0.25	0.12	0	1	704
Beds in Trust 400–549	0.17	0.37	0.34	0.17	0	1	704
Beds in Trust 550–699	0.20	0.40	0.36	0.18	0	1	704
Beds in Trust 700–849	0.15	0.36	0.31	0.19	0	1	704
Beds in Trust 850–999	0.14	0.35	0.30	0.19	0	1	704
Beds in Trust 1000–1149	0.10	0.30	0.23	0.19	0	1	704
Beds in Trust 1150+	0.17	0.37	0.34	0.15	0	1	704
Count of staff	4563	2340	2350	420.3	1344	13,190	704
% of staff doctors	12.86	2.33	2.22	0.79	5.39	25.87	704
% of staff nurses or midwives	31.13	2.82	2.75	0.84	22.33	42.45	704
% of staff managers	2.27	0.82	0.78	0.26	0.57	5.67	704
Teaching Trust	0.18	0.39	0.39	0.00	0	1	704
Foundation Trust	0.49	0.50	0.49	0.10	0	1	704
Number of sites in Trust	2.70	2.18	2.00	0.82	1.00	15.00	704
Equivalent number of Rivals	4.18	2.71	2.77	0.00	1.00	13.60	704
*Local Area Characteristics*							
Total Population within 30 km (100 000s)	28.41	28.87	30.25	0.66	1.34	99.75	704
% of population aged 65+ within 30 km	16.81	3.26	3.30	0.53	11.20	25.30	704
Income deprivation rank 1000s	16.63	2.77	2.76	0.00	9.40	20.97	704
Average distance to nearest GP for population within 30 km	1.56	0.58	0.58	0.00	0.81	3.43	704
Market Forces Factor	1.08	0.07	0.07	0.004	1.00	1.32	704

a FFT score quarterly for 2013/14, FFT recommendation rate quarterly for 2014/15, A&E waiting times: quarterly 2010/11–2014/15.

b 2010/11–2013/14.

Hospitals have on average 827 beds, with a minimum of 259 beds and a maximum of 2478 beds. 7.7% are small (less than 400 beds). The proportions of hospitals in the other bed categories are between 9.8% (hospitals with 1000–1149 beds) and 19.9% (hospitals with 550–699 beds).

There are on average 4563 hospital staff of whom 13% are doctors, 31% are nurses and midwifes, and 2.3% are managers. 37% of patients are emergencies, 43% are male, and 63% are 45 years old or older. Each hospital (Trust) has on average 2.7 sites. 18% of hospitals are Teaching Trusts, and 49% have Foundation Trust status. Each hospital competes on average with (an equivalent number of) 4.2 hospitals within a catchment area of 30 km.

There is on average a total population of 2.8 million within a catchment area of 30 km from the hospital. On average 17% of the population within this catchment is older than 65 years and their average distance to a GP is 1.6 km. The MFF is an index with an average of 1.08 and a standard deviation of 0.07, a minimum of 1 (reflecting the hospital with the lowest unavoidable cost which receives the base tariff for a HRG) and a maximum of 1.32. The mean LSOA level rank of local income deprivation in all hospital catchment areas is 16,630, compared with the median national rank of 16,422. The mean rank for a hospital catchment area ranges from 9397 (29th percentile) to 20,970 (64th percentile) of the national distribution of ranks, with higher ranks indicating higher income deprivation.

Table 1 also reports the *overall* standard deviation (across all hospitals and years), the *between* standard deviation (variation across hospitals) and the *within* standard deviation (variation over time within hospitals). For most of the quality measures the variation within hospitals is roughly as great as the variation between them. By contrast, there is less variation within rather than across hospitals in patient, hospital and local area characteristics. This is especially so for hospital size.

### 4.2 Regression results

Results for Model 1, which includes only bed size categories, patient characteristics and year or quarter dummies, are in Table 2a, Table 2b, Table 2ca-2c In Table 2a (first column) having 400 or more beds is associated with higher overall SHMI mortality, though the association is statistically significant at the 5% level for only one of the six beds-size categories. By contrast, in column 2, larger hospitals have *lower* AMI mortality than the baseline small hospital category and the differences are statistically significant for the largest five size categories, with hospitals over 550 beds having AMI mortality which is 1.5–1.7 percentage points (pp) lower than in small hospitals with under 400 beds. Since mean AMI mortality is 5.3%, a difference of 1.5pp is 1.5/5.3 = 0.28 = 28% of the mean mortality. Non-elective mortality is higher by 0.27–0.37 percentage points in hospitals with over 850 beds relative to those with under 400 beds (column 3). Compared with mean non-elective mortality of 3.7%, a difference 0.27pp is 0.27/3.7 = 0.073 = 7.3% of the mean mortality. There is no statistically significant association of size with hip fracture or stroke mortality (columns 4 and 5).

**Table 2a:** Mortality rates (model 1).

	SHMI Overall Mortality		AMI (heart attack) Mortality Rate		Non-Elective Mortality Rate		Hip Fracture Mortality Rate		Stroke Mortality Rate	
	coeff	p-value	coeff	p-value	coeff	p-value	coeff	p-value	coeff	p-value
Bed Categories										
Beds 400–549	0.140	0.933	−0.632	0.363	0.095	0.371	−0.511	0.110	0.686	0.231
Beds 550–699	2.513*	0.097	−1.528**	0.018	0.011	0.909	−0.442	0.148	0.012	0.981
Beds 700–849	4.130***	0.008	−1.775***	0.006	0.086	0.378	−0.436	0.170	−0.511	0.328
Beds 850–999	2.389	0.128	−1.514**	0.020	0.268**	0.011	−0.361	0.249	−0.371	0.511
Beds 1000–1149	1.976	0.214	−1.549**	0.017	0.372***	0.001	−0.420	0.218	0.262	0.672
Beds 1150+	0.452	0.768	−1.759***	0.005	0.280***	0.005	−0.465	0.104	−0.687	0.210
Constant	153***	0.000	−17.00***	0.009	−5.160***	0.004	−3.879	0.484	8.024	0.468
Observations	704		495		686		665		678	
R^2^	0.454		0.108		0.318		0.159		0.094	

Notes: * = p < 0.1, ** = p < 0.05, *** = p < 0.01. Results from pooled OLS. Robust standard errors are clustered at Trust level. Baseline: < 400 beds, % Age 60–74, year 2010–11. Year dummies and casemix controls (age in year bands, % male, % of emergency admissions) are not reported.

In Table 2b there is no association between size and infection rates (columns 1, 2). Costs are lower in all hospitals compared to the baseline small hospitals (column 3), but A&E waiting times are higher. There is no statistically significant association between size and any of the patient experience measures (Table 2c) except that hospitals with 700–999 beds have higher cleanliness scores by 1.25 percentage points than the baseline small hospitals (column 1), which accounts for only 1.4% (1.25/87.39) of the mean cleanliness outcome.

**Table 2b:** Infections, hospital costs and A&E waiting times (model 1).

	*MRSA infections rate*		*C-Difficile infections rate*		*Reference Cost Index*		*A&E waiting times > 4 h*	
	coeff	p-value	coeff	p-value	coeff	p-value	Coeff	p-value
Bed Categories								
Beds 400–549	0.040	0.826	0.587	0.677	−2.049*	0.083	0.069	0.757
Beds 550–699	−0.293*	0.073	−1.554	0.203	−5.439***	0.000	0.874***	0.000
Beds 700–849	−0.190	0.270	−0.060	0.963	−3.320***	0.003	1.166***	0.000
Beds 850–999	0.048	0.785	0.738	0.576	−3.058***	0.006	1.119***	0.000
Beds 1000–1149	0.193	0.331	0.304	0.817	−2.821**	0.018	2.079***	0.000
Beds 1150+	0.039	0.823	0.300	0.807	−2.218**	0.044	1.311***	0.000
Observations	702		704		704		2235	
R^2^	0.243		0.365		0.251		0.316	

Notes: * = p < 0.1, ** = p < 0.05, *** = p < 0.01. Results from pooled OLS. Robust standard errors are clustered at Trust level. Baseline: < 400 beds, % Age 60–74, year 2010–11. Year dummies for infections rates and Reference Cost Index not reported; similarly, quarterly dummies for A&E waiting times not reported. Casemix controls (age in year bands, % male, % of emergency admissions) also not reported.

**Table 2c:** Patient experience (model 1).

	*Cleanliness Score*		*Involvement Score*		*Dignity Score*		*FFT recommendation rate*	
	coeff	p-value	coeff	p-value	Coeff	p-value	coeff	p-value
Bed Categories								
Beds 400–549	0.643	0.141	0.104	0.853	0.282	0.496	−0.706	0.215
Beds 550–699	0.474	0.284	−0.466	0.398	0.537	0.179	−0.095	0.869
Beds 700–849	1.246***	0.008	−0.814	0.147	0.521	0.215	0.596	0.272
Beds 850–999	0.713*	0.092	−0.143	0.796	0.348	0.367	−0.690	0.260
Beds 1000–1149	0.770	0.144	−0.289	0.619	0.443	0.297	−0.284	0.648
Beds 1150+	0.858*	0.053	−0.319	0.572	0.249	0.538	−0.199	0.726
Constant	120***	0.000	114***	0.000	128***	0.000	87.82***	0.000
Observations	568		568		568		532	
R^2^	0.239		0.303		0.219		0.102	

Notes: * = p < 0.1, ** = p < 0.05, *** = p < 0.01, Results from pooled OLS. Robust standard errors are clustered at Trust level. Baseline: < 400 beds, % Age 60–74, year 2010–11. Year dummies for Cleanliness, Involvement and Dignity Score for 2011/12–2013/14 not reported; similarly, quarterly dummies for FFT recommendation rate in 2014/15 not reported (baseline quarter 1 of 2014/15). Casemix controls (age in year bands, % male, % of emergency admissions) also not reported.

The results for the simple models suggests that, after controlling only for casemix, it is not the case that patients attending small hospitals have generally lower quality relative to patients attending larger hospitals. The only exception is AMI mortality, which is higher in small hospitals with under 400 beds relative to some of the larger size categories.

In Table 3a, Table 3b, Table 3ca-3c we report the results for Model 2, which additionally contains hospital and local area characteristics and random hospital effects. The Breusch-Pagan tests indicate that this random effects specification is better than a pooled OLS specification with the same explanatories.

AMI mortality is again lower, by about 0.95–1.7 percentage points, in hospitals with more than 550 beds compared with those with under 400 beds (second column of Table 3a). There are no statistically significant differences between small hospitals and those in larger size categories for SHMI, overall non-elective mortality, hip fracture and stroke mortality (first, third, fourth and fifth column in Table 3a), infection rates (first and second column of Table 3b), waiting times for emergency care (fourth column of Table 3b) or patient experience (Table 3c).

**Table 3a:** Mortality rates (model 2).

	*Overall mortality SHMI*		AMI (heart attack) Mortality Rate		Non-Elective Mortality Rate		Hip Fracture Mortality Rate		Stroke Mortality Rate	
	coeff	p-value	coeff	p-value	coeff	p-value	Coeff	p-value	coeff	p-value
Bed Categories										
Beds 400–549	1.117	0.579	−0.459	0.378	0.114	0.340	−0.227	0.536	0.532	0.307
Beds 550–699	1.430	0.490	−0.950*	0.084	0.052	0.685	−0.162	0.661	−0.099	0.859
Beds 700–849	1.526	0.482	−1.338**	0.030	0.047	0.734	−0.367	0.345	−0.567	0.337
Beds 850–999	1.179	0.597	−1.413**	0.022	0.169	0.245	−0.222	0.597	−0.916	0.183
Beds 1000–1049	0.065	0.977	−1.627**	0.012	0.138	0.354	−0.433	0.304	0.050	0.949
Beds 1150+	0.018	0.993	−1.751***	0.010	0.143	0.377	−0.185	0.667	−0.811	0.229
Patient Characteristics										
% Age 0–14	0.562*	0.087	0.049	0.690	0.012	0.702	0.006	0.944	0.209	0.213
% Age 15–29	0.649	0.142	0.124	0.408	0.070*	0.060	0.081	0.456	0.038	0.888
% Age 30–44	−0.239	0.638	0.033	0.886	−0.028	0.551	−0.070	0.594	0.189	0.495
% Age 45–59	0.417	0.517	0.083	0.707	0.082	0.168	−0.046	0.786	0.274	0.412
% Age 75–89	1.338***	0.009	0.100	0.612	0.071	0.103	0.149	0.280	0.157	0.559
% Age 90+	−2.274**	0.027	0.651*	0.076	−0.206***	0.009	−0.775***	0.001	0.193	0.643
% Male	0.171	0.577	0.000	0.998	0.038*	0.087	0.060	0.452	0.071	0.541
% Admissions Emergencies	−0.150	0.126	−0.008	0.790	−0.002	0.774	0.040*	0.081	−0.031	0.471
Trust Characteristics										
% staff doctors	0.514***	0.003	0.005	0.960	0.030*	0.087	−0.049	0.257	0.101	0.311
% staff nurses or midwives	0.481**	0.018	0.103	0.139	0.028**	0.038	−0.026	0.454	−0.080	0.219
% staff managers	−0.058	0.903	0.288*	0.082	−0.020	0.622	−0.032	0.801	−0.286	0.188
Teaching	−5.938***	0.001	0.321	0.490	0.183	0.279	−0.481	0.234	−1.563**	0.012
Foundation Trust	0.010	0.992	−0.142	0.534	−0.061	0.387	−0.117	0.513	−0.297	0.360
Two Sites	1.368*	0.099	0.433	0.157	−0.022	0.770	−0.212	0.395	0.202	0.626
Three Sites	0.934	0.358	0.504	0.116	0.077	0.371	0.279	0.336	0.235	0.562
Four + Sites	1.156	0.331	0.469	0.198	0.016	0.870	0.270	0.368	0.172	0.722
Equivalent Rivals	−0.675*	0.070	0.203	0.121	−0.028	0.322	0.091	0.279	0.092	0.491
Local Area Characteristics										
Pop within 30 km (100 000s)	−0.144**	0.013	−0.023	0.209	0.001	0.831	0.003	0.834	0.028	0.256
% pop aged 65+ within 30 km	−0.170	0.598	−0.157	0.154	0.020	0.426	−0.053	0.425	0.089	0.500
Income deprivation rank (1000s)	0.244	0.391	0.140*	0.083	0.046**	0.029	0.024	0.693	−0.151	0.217
Average distance to nearest GP	−5.286***	0.003	0.131	0.699	−0.282**	0.023	0.159	0.647	−0.809	0.205
Market Factor Forces	−35.67	0.129	0.848	0.912	0.962	0.612	1.417	0.798	−21.76*	0.051
Constant	90.95**	0.031	−6.142	0.635	−3.314	0.323	3.584	0.718	29.53	0.158
Observations	704		495		686		665		678	
Breusch-Pagan Test	351***	0.000	22.28***	0.000	233***	0.000	78.94***	0.000	96.56***	0.000
R^2^ (overall)	0.530		0.152		0.385		0.181		0.113	

Notes: * = p < 0.1, ** = p < 0.05, *** = p < 0.01. Results from models with random hospital effects. Robust standard errors are clustered at Trust level. Baseline: < 400 beds, % Age 60–74, year 2010–11, non-Teaching, non-FT, single Site. Year dummies are not reported. Breusch-Pagan test H^o^: OLS residuals do not contain individual hospital effects.

**Table 3b:** Infections, hospital costs and A&E waiting times (model 2).

	*MRSA infections rate*		*C-Difficile infections rate*		*Reference Cost Index*		*A&E waiting times > 4 h*	
	coeff	p-value	Coeff	p-value	coeff	p-value	coeff	p-value
Bed Categories								
Beds 400–549	0.064	0.731	1.367	0.342	−0.443	0.655	−0.270	0.418
Beds 550–699	−0.285*	0.077	−0.201	0.890	−2.389**	0.030	0.353	0.411
Beds 700–849	−0.221	0.214	1.752	0.255	−1.174	0.374	0.557	0.263
Beds 850–999	−0.121	0.547	0.763	0.641	−0.998	0.460	0.753	0.184
Beds 1000–1049	0.090	0.672	1.302	0.422	−1.144	0.428	0.783	0.231
Beds 1150+	−0.180	0.369	0.878	0.600	−1.201	0.408	0.918	0.168
Patient Characteristics								
% Age 0–14	0.004	0.923	0.002	0.995	−0.186	0.489	−0.041	0.783
% Age 15–29	0.008	0.866	−0.451	0.310	0.096	0.783	−0.087	0.621
% Age 30–44	0.063	0.268	0.137	0.786	−0.450	0.259	0.131	0.473
% Age 45–59	−0.011	0.889	0.131	0.852	0.528	0.247	−0.543**	0.024
% Age 75–89	−0.039	0.553	−0.200	0.725	−0.645*	0.091	0.069	0.779
% Age 90+	0.098	0.420	−0.690	0.550	−0.276	0.710	−0.782	0.199
% Male	0.054**	0.041	0.152	0.577	0.278	0.247	0.110	0.365
% Admissions Emergencies	0.021**	0.038	−0.016	0.870	−0.004	0.952	0.028	0.464
Trust Characteristics								
% staff doctors	0.086***	0.002	0.164	0.414	−0.362**	0.018	−0.015	0.880
% staff nurses or midwives	0.020	0.211	0.077	0.659	0.125	0.293	−0.001	0.988
% staff managers	−0.016	0.752	0.530	0.279	−0.570	0.144	−0.037	0.841
Teaching	−0.075	0.662	2.204	0.147	0.760	0.516	0.294	0.618
Foundation Trust	−0.032	0.687	0.036	0.965	−2.383***	0.002	−0.854***	0.004
Two Sites	0.152	0.214	−1.700*	0.067	0.035	0.967	0.305	0.731
Three Sites	0.246*	0.057	−1.677	0.122	0.742	0.387	−0.056	0.941
Four + Sites	0.179	0.239	−0.689	0.588	−0.088	0.926	0.157	0.820
Equivalent Rivals	0.044	0.117	0.011	0.971	0.217	0.410	−0.170	0.171
Local Area Characteristics								
Pop within 30 km (100 000s)	0.004	0.456	0.077*	0.087	0.007	0.863	−0.170	0.171
% pop aged 65+ Within 30 km	0.018	0.465	0.506*	0.066	0.319	0.153	−0.008	0.648
Income deprivation rank (1000s)	−0.009	0.757	−0.148	0.536	0.237	0.339	−0.057	0.677
Average distance to nearest GP of pop (30 km)	−0.081	0.507	−1.353	0.337	1.369	0.302	−0.066	0.552
MFF	−2.781	0.301	−45.54**	0.014	13.39	0.418	−1.319	0.869
Constant	−0.734	0.863	67.739**	0.045	77.64***	0.010	12.67	0.391
Observations	702		704		704.0		2235	
Breusch-Pagan Test	9.760***	0.001	65.51***	0.000	317.7***	0.000	1036***	0.000
R^2^ (overall)	0.275		0.393		0.286		0.327	

Notes: * = p < 0.1, ** = p < 0.05, *** = p < 0.01, robust standard errors are clustered at Trust level. Baseline: < 400 beds, % Age 60–74, year 2010–11, non-Teaching, non-FT, single Site. Year dummies for infections rates and Reference Cost Index not reported; similarly, quarterly dummies for A&W waiting times not reported (baseline quarter 1 of 2010–11). Breusch-Pagan test H^o^: OLS residuals do not contain individual hospital effects.

**Table 3c:** Patient experience (Model 2).

	*Cleanliness Score*		*Involvement Score*		*Dignity Score*		*FFT recommendation*	
	coeff	p	Coeff	p	coeff	p	Coeff	P
Bed Categories								
Beds 400–549	−0.246	0.620	−0.461	0.394	−0.198	0.590	0.768	0.352
Beds 550–699	−0.374	0.539	−0.914	0.156	0.158	0.713	1.452*	0.089
Beds 700–849	0.498	0.470	−0.965	0.158	0.387	0.421	1.639*	0.072
Beds 850–999	−0.121	0.860	0.264	0.702	0.308	0.490	0.410	0.787
Beds 1000–1049	−0.090	0.901	−0.384	0.582	0.083	0.857	0.761	0.576
Beds 1150+	0.254	0.723	−0.066	0.923	0.246	0.613	1.305	0.239
Patient Characteristics								
% Age 0–14	−0.167	0.116	−0.013	0.917	−0.095	0.374	−0.154	0.464
% Age 15–29	−0.184	0.175	−0.203	0.119	−0.293**	0.020	0.098	0.713
% Age 30–44	−0.250*	0.096	0.035	0.841	0.013	0.932	−0.418	0.155
% Age 45–59	0.149	0.461	0.197	0.417	0.008	0.970	−0.050	0.898
% Age 75–89	−0.343**	0.028	−0.251	0.154	−0.244	0.120	−0.104	0.813
% Age 90+	0.124	0.741	0.646*	0.096	0.195	0.525	−0.006	0.993
% Male	−0.116	0.225	−0.068	0.510	−0.049	0.493	0.100	0.499
% Admissions Emergencies	−0.004	0.909	−0.083**	0.017	−0.033	0.240	−0.171**	0.034
Trust Characteristics								
% staff doctors	−0.171**	0.028	−0.243***	0.007	−0.190***	0.005	−0.152*	0.098
% staff nurses or midwives	0.004	0.940	−0.096	0.116	0.004	0.929	0.109	0.276
% staff managers	−0.384**	0.033	0.333*	0.051	0.230*	0.093	−0.138	0.721
Teaching	1.041*	0.079	1.154**	0.027	1.222***	0.005	−0.587	0.521
FT	0.527*	0.064	1.109***	0.000	0.793***	0.001	−0.018	0.973
Two Sites	−0.336	0.350	−0.328	0.384	−0.169	0.558	−0.049	0.945
Three Sites	−0.804**	0.042	−0.672	0.110	−0.710**	0.027	−1.797	0.109
Four + Sites	−0.331	0.439	−0.238	0.566	−0.143	0.654	−0.463	0.598
Equivalent Rivals	0.147	0.251	0.173	0.132	0.165*	0.067	0.069	0.697
Local Area Characteristics								
Pop within 30 km (100 000s)	−0.034*	0.073	−0.007	0.667	−0.021*	0.085	0.069	0.697
% pop aged 65+ Within 30 km	0.230*	0.051	0.233*	0.072	0.179*	0.094	0.008	0.971
Income deprivation rank (1000s)	−0.032	0.749	−0.140	0.149	−0.142**	0.037	−0.424***	0.004
Av distance to nearest GP of pop (30 km)	−0.969*	0.068	0.582	0.335	−0.363	0.425	−0.724	0.468
Market Factor Forces	4.830	0.516	−2.418	0.735	−0.927	0.876	−16.73	0.221
Constant	100.5***	0.000	83.55***	0.000	102.2***	0.000	128.0***	0.000
Observations	568		568		568		532	
Breusch-Pagan Test	342***	0.000	109***	0.000	95.25***	0.000	358.0***	0.000
R^2^ (overall)	0.353		0.470		0.409		0.194	

Notes: * = p < 0.1, ** = p < 0.05, *** = p < 0.01, robust standard errors are clustered at Trust level. Baseline: < 400 beds, % Age 60–74, year 2010–11, non-Teaching, non-FT, single Site. Year dummies for Cleanliness, Involvement and Dignity Score for 2011/12–2013/14 not reported; similarly, quarterly dummies for FFT recommendation rate in 2014/15 not reported (baseline quarter 1 of 2014/15). Breusch-Pagan test H^o^: OLS residuals do not contain individual hospital effects.

The results for AMI are based on a relatively small number of observations of AMI mortality in hospitals in the lowest size category. For example, there are SHMI data for 18 hospitals (54 observations) which have less than 400 beds. By contrast there are AMI mortality data for only 12 hospitals and 18 hospital-year observations with less than 400 beds. This may be because treatment of AMI patients is more concentrated within larger hospitals and AMI mortality data is reported only if a hospital has at least 6 deaths in the year.

Some of the additional control variables in Model 2 and reported in Table 3a, Table 3b, Table 3ca-3b are associated with quality. Teaching hospitals have lower SHMI (by 5.9pp or 5.9% given a SHMI mean of 100) and stroke mortality (by 1.56pp or 1.56/17 = 9.2%) and higher satisfaction from patients involvement and being treated with dignity by 1–1.2pp (or 1.2–1.6%). Foundation Trusts also have higher patient satisfaction in these dimensions with smaller magnitude relative to the teaching coefficient. Hospitals with three sites have lower satisfaction in two domains relative to hospitals with one site. Hospitals serving populations with higher income deprivation have higher mortality for non-elective treatments and lower satisfaction in the dignity domain. A higher proportion of doctor staff and of nurses or midwives is associated with higher overall and non-elective mortality. A higher proportion of doctor staff is also associated with lower satisfaction. This is possibly because hospitals with lower quality are more likely to recruit a higher proportion of medical staff to address the lower quality. With few exceptions patient and local characteristics are generally not associated with quality at the 5% significance level.

The within-between specification (Tables B.1–3, Online Appendix B) separately investigates associations between quality and variations in the explanatories within and between hospitals. Because of the relatively small variation in the numbers of beds within hospitals, the within-hospital time-varying beds categories are not significantly associated with AMI mortality or any other measures of quality. The estimated coefficients on the means of the time-varying size variables pick up the association of unobserved time-invariant hospital factors with quality and cannot be interpreted as evidence of an association of average size and quality across hospitals.

As a robustness check we re-estimated Model 2 restricting the sample to single-site hospitals. The samples drops to about one third of the original sample (one fourth for AMI). Given that most large hospitals have more than one site, we use a single size category for hospitals with more than 700 beds. The results (Appendix C) are broadly in line with Table 3a, Table 3b, Table 3ca-3c Hospitals with less than 400 beds are not associated with lower quality except for AMI mortality, but this is only significant at the 10% level. In Appendix D, we also provide the results for the 2013-14 Friends and Family Test measured as a score (rather than as a rate in 2014–15). Again, small hospitals are not associated with lower quality. Finally, in Appendix E we compare the marginal effects of the hospital size categories from linear models with those from a fractional logit regressions. The results are very similar.

## 5 Discussion

Our analysis suggests that, from a *patient* perspective, patients who attend small hospitals with less than 400 beds do not experience lower quality relative to patients attending larger hospitals, with the possible exception of AMI mortality. Thus, from an equity perspective, we find no strong evidence of systematic differences in quality of care by patients who live closer to smaller hospitals, due to work or other reasons, compared to patients who live close to large hospitals (e.g. in urban areas).

Moreover, small hospitals do not generally exhibit lower quality after controlling for a range of characteristics of the hospital and of the catchment of the area they serve. We find that being a teaching hospital is a marker of good quality in several clinical and non-clinical quality domains. Foundation Trusts also have higher patient satisfaction possibly because of their higher degree of autonomy. Although teaching hospitals and Foundation Trusts tend to be larger, this is not responsible for the (lack of) association between small size and quality: the quality of relatively large hospitals without teaching or Foundation Trust status does not differ systematically from that in small hospitals. Hospitals with more beds are more likely to have more sites and therefore possibly be more difficult to manage; and we find that having more sites is associated with lower patient satisfaction (but not with lower clinical quality). Nevertheless, controlling for the number of sites does not alter the associations between small hospital size and quality.

Hospitals serving populations with higher income deprivation have lower quality in some domains (higher mortality rates for non-elective treatments and lower satisfaction in the dignity domain), perhaps because of the additional demand pressure in more deprived areas. However, this is not the case where such demand pressures are measured as the proportion of over 65 years old. We did not find that supply factors, such as local input prices, are systematically associated with quality. One possible explanation is that hospital tariffs are adjusted to take into account such unavoidable cost differences across hospitals, and this adjustment is effective in avoiding quality differences which may arise due to different costs.

With respect to our general findings, the exception is AMI mortality, where larger hospitals have about 1.5 percentage points lower mortality risk (recall average AMI mortality rate is 5.3%) and this holds after controlling for hospital and catchment area characteristics. This association could be due to a lack of specialised personnel, and greater difficulty in recruiting clinical staff. Small hospitals are more likely to be located in rural areas. Longer travel time before hospital treatment can increase mortality given the importance of early stabilization (Dhakam and Khalid, 2008) for AMI patients. Small hospitals may have a shortage of equipment required for core life-saving treatments for AMI patients, such as percutaneous coronary intervention or coronary artery bypass grafting (CABG). This particularly holds for CABG surgery, which is only provided in about 50 generally large NHS hospitals.

As noted earlier, there are fewer observations of small hospitals with reported AMI mortality than for other quality measures so that the estimated association may be misleading. Even if they are due to an effect of size on AMI mortality, specific policies to improve staffing and equipment for care of AMI patients in small hospitals may be a more appropriate response, rather than merging and closing small hospitals, given the lack of evidence of any effect of size on other dimensions of quality.

Although we have used a comprehensive set of quality measures, future work could extend the analysis in a number of ways. First, since hospital size appears to change only to a limited extent over short periods, future work could extend the time period covered to examine whether changes in hospital size, perhaps due to restructuring, over time are associated with changes in quality within hospitals. Second, additional overall hospital quality and responsiveness measures should be included in the analysis, including, for example, emergency readmission rates, Care and Quality Commission hospital ratings, cancelled operation rates, and adverse events.

## 6 Conclusion

Our analysis does not provide any strong support for the proposition that small NHS acute hospitals in England provide generally lower quality to their patients. One explanation for our findings is that small NHS hospitals in England are larger than small hospitals in other OECD countries (Vaughan et al., 2018): for example, France has over 2000 hospitals (Choné, 2017), Germany had about 1980 hospitals in 2014 (Kifmann, 2017). Thus small English hospitals may be large enough to have exploited any scale economies in the provision of quality. One implication is that reconfigurations involving mergers and closures of small hospitals run the risk of reducing patient access to hospitals, with no clear offsetting benefits in terms of higher quality.

## Author contribution

James Gaughan: Methodology, regression analysis, data collection, writing. Luigi Siciliani: conceptualisation, Methodology, writing, coordination. Hugh Gravelle: conceptualisation, Methodology, writing. Giuseppe Moscelli: Methodology, data, writing.

## Declaration of competing interest

None.
