# Supplementary material for: Do small hospitals have lower quality? Evidence from the English NHS
Source: Soc Sci Med. 2020 Nov;265:113500. doi: 10.1016/j.socscimed.2020.113500 (PMC7768184; doi:10.1016/j.socscimed.2020.113500)

# Online Appendix

# Appendix A: Data Appendix

# Table A1: Description of variables

| Variable name | Description | Frequency | Source |
| --- | --- | --- | --- |
| SHMI (Overall mortality) | Observed/expected mortality within 30 days, centred around 100. Expected mortality calculated from national average rates by age, gender and diagnosis category in the same year. | Annual | NHS Digital Indicator Portal |
| AMI, Non-elective, Hip fracture or Stroke mortality rate | Indirectly standardised mortality rate: (observed deaths/expected deaths) times crude death rate. Expected mortality calculated from national average rates by age and gender, using the national average mortality rates from 2010/11. | Annual | NHS Digital Indicator Portal |
| Rates of MRSA, C-Difficile infections per 100,000 bed days | The number of MRSA, C-Difficile infections per 100,000 bed days of patients aged 2+ and ‘apportioned to a trust’. Infections are apportioned to a trust if patient location was within an acute trust (hospital) and had been admitted as an inpatient, day case or for emergency assessment at least three days previously. | Annual | NHS Digital |
| Reference Cost Index | Observed/expected costs, centred around 100. Expected costs calculated from national average costs by case-mix categories. | Annual | PHE Reports |
| FFT score | Proportion of respondents extremely likely to recommend minus proportion who would not recommend. | Quarterly | NHS England |
| FFT recommendation rate | Percentage of patients who would recommend the Trust to friends and family (available only for 2014-15) | Quarterly | NHS England |
| Mean Score for Cleanliness/Involvement  /Dignity Question | Mean scores, range 0-100, constructed from the inpatient survey. Surveys were delivered by post from August of the survey year to January of the following year. Responses are weighted by age group, sex and admission route. | Annual | CQC, further prepared by the King’s Fund |
| A&E waiting times > 4 hours | Percentage of patients who spent at least 4 hours in an Accident and Emergency Department from arrival to disposal via admission, discharge or death. | Quarterly | NHS England |
| % Patient Age 0-14, 15-29, 30-44, 45-59, 60-74, 75-89, 90+ | Percentage of patients in a given age group. | Annual | HES aggregate figures published by NHS Digital |
| % Patients Male | Percentage of patients within a hospital who are male. | Annual |  |
| % Admissions Emergencies | Percentage of admissions within a hospital which are emergencies. | Annual |  |
| Beds in hospital | Mean number of overnight and day case beds. This data is reported daily and published quarterly. The mean of quarterly figures is calculated to aggregate to the annual level. | Annual | NHS England Available and Occupied Beds |
| % of staff doctors, nurses or midwives, managers | Percentage of staff within each category out of the total number of staff. Data are reported on the last day of each month and the mean of these values taken to aggregate to the annual level. | Annual | ESR collated by NHS Digital |
| Teaching Trust | = 1 if a Trust is a Teaching Trust | No change over time | Patient Safety Incident Reports |
| Foundation Trust | = 1 if a Trust has Foundation Trust status in a given year | Annual | Directory of Foundation Trusts |
| Number of sites in Trust | Count of hospital sites within a Trust | Annual | HES |
| Equivalent Rivals within 30km from Predicted HHI | Equivalent number of rivals within 30km from predicted HHI | Single snapshot | HES |
| Total population within 30km (100,000s) | The number of people living in LSOAs with centroids within 30km of the headquarters of the Trust. | Annual | ONS mid-year population estimates |
| % of pop aged 65+ within 30km | The percentage of population living in LSOAs with centroids within 30km of the headquarters of the Trust who are aged 65+ | Annual | ONS mid-year population estimates |
| Income deprivation rank (1,000s) | Mean income deprivation rank of LSOAs with centroids within 30km of the Trust headquarters. Ranks of individual LSOAs range from 1-32,844. | Single snapshot | IMD 2015 |
| Average distance to nearest GP of population within 30km | Mean distance to the nearest GP in LSOAs with centroids within 30km of the Trust headquarters. | Single snapshot | IMD 2015 |
| Market Forces Factor | Adjustment factor applied to base tariffs paid to Trusts. The factor reflects unavailable variation in costs of land and labour in the Trust. | Annual | National tariff of payments |

Notes: AMI = Acute Myocardial Infarction; CQC = Care Quality Commission; ESR = Electronic Staff Record; HES = Hospital Episode Statistics; IMD = Index of Multiple Deprivation; LSOA = Lower Super Output Area; MRSA = Methicillin-resistant Staphylococcus aureus; NHS = National Health Service; ONS = Office of National Statistics; PHE = Public Health England; SHMI = Standardised Hospital Mortality Indicator.

# Table A2: Summary of OPCS codes in non-elective mortality indicator

| OPCS chapter letter – name | Number of subchapters included |
| --- | --- |
| A – Nervous System | 9 |
| C – Eye | 1 |
| D – Ear | 1 |
| E – Respiratory Tract | 7 |
| G – Upper Digestive System | 37 |
| H – Lower Digestive System | 20 |
| J – Other Abdominal Organs, Principally Digestive | 28 |
| K – Heart | 18 |
| L – Arteries and Veins | 41 |
| M – Urinary | 5 |
| N – Male Genital Organs | 1 |
| Q – Upper Female Genital Tract | 2 |
| S – Sin | 2 |
| T – Soft Tissue | 16 |
| V – Bones and Joints of Skull and Spine | 1 |
| W – Other Bones and Joints | 18 |
| X – Miscellaneous Operations | 8 |

Notes: OPCS = Operations and Procedure Codes.

# Appendix B: Within-between specification

# Table B1: Mortality (Within-between specification)

|  | Overall mortality  SHMI | | | AMI  Mortality Rate | | | Non-Elective  Mortality Rate | | | Hip Fracture  Mortality Rate | | Stroke  Mortality Rate | |
| --- | --- | --- | --- | --- | --- | --- | --- | --- | --- | --- | --- | --- | --- |
|  | b | p | b | | p | b | | p | b | | p | b | p |
| Deviations (dev) and time invariant (inv) variables | | | | | | | | | | | | | |
| Bed Categories | | | | | | | | | | | | | |
| Beds 400-549 (dev) | 2.292 | 0.338 | 0.263 | | 0.610 | 0.219 | | 0.227 | -0.133 | | 0.789 | 0.818 | 0.103 |
| Beds 550-699 (dev) | 2.568 | 0.365 | 1.110 | | 0.197 | 0.251 | | 0.217 | -0.078 | | 0.910 | 1.251 | 0.116 |
| Beds 700-849 (dev) | 2.545 | 0.403 | 0.972 | | 0.308 | 0.214 | | 0.370 | -0.377 | | 0.581 | 1.337 | 0.183 |
| Beds 850-999 (dev) | 3.245 | 0.311 | 0.299 | | 0.739 | 0.328 | | 0.173 | 0.092 | | 0.901 | 1.253 | 0.314 |
| Beds 1000-1049 (dev) | 2.187 | 0.533 | -0.092 | | 0.933 | 0.218 | | 0.389 | -0.074 | | 0.919 | 2.610** | 0.048 |
| Beds 1050+ (dev) | 2.358 | 0.546 | 0.078 | | 0.945 | 0.274 | | 0.300 | 0.524 | | 0.504 | 2.189 | 0.121 |
| Patient Characteristics | | | | | | | | | | | | | |
| % Age 0-14 (dev) | 0.925* | 0.088 | 0.276 | | 0.255 | 0.075 | | 0.156 | -0.232 | | 0.124 | 0.364 | 0.174 |
| % Age 15-29 (dev) | 0.769 | 0.243 | 0.112 | | 0.720 | 0.040 | | 0.518 | -0.152 | | 0.469 | 0.926** | 0.020 |
| % Age 30-44 (dev) | 0.023 | 0.982 | 0.274 | | 0.517 | 0.020 | | 0.805 | -0.079 | | 0.741 | -0.068 | 0.868 |
| % Age 45-59 (dev) | 0.046 | 0.954 | 0.164 | | 0.657 | 0.106 | | 0.199 | -0.318 | | 0.209 | 0.885* | 0.062 |
| % Age 75-89 (dev) | 1.540** | 0.030 | 0.244 | | 0.505 | 0.142** | | 0.045 | 0.023 | | 0.914 | 0.085 | 0.821 |
| % Age 90+ (dev) | -1.315 | 0.370 | 1.517** | | 0.019 | -0.131 | | 0.256 | -1.164*** | | 0.002 | 0.904 | 0.235 |
| % Male (dev) | 0.522 | 0.268 | -0.102 | | 0.463 | 0.034 | | 0.389 | 0.151 | | 0.201 | 0.519* | 0.055 |
| % Admissions  Emergencies (dev) | -0.441** | 0.011 | -0.021 | | 0.615 | -0.006 | | 0.600 | 0.033 | | 0.449 | -0.157** | 0.020 |
| Trust Characteristics | | | | | | | | | | | | | |
| % Staff doctors (dev) | 0.717*** | 0.000 | 0.124 | | 0.475 | 0.033* | | 0.074 | -0.096 | | 0.104 | 0.004 | 0.968 |
| % Staff nurses or  midwives (dev) | 0.648* | 0.076 | -0.009 | | 0.958 | 0.011 | | 0.552 | -0.044 | | 0.489 | -0.407*** | 0.006 |
| % Staff managers (dev) | 0.617 | 0.426 | 0.228 | | 0.406 | -0.026 | | 0.657 | 0.077 | | 0.705 | -0.076 | 0.866 |
| Teaching (inv) | -3.048 | 0.174 | 0.262 | | 0.636 | 0.348* | | 0.080 | -0.155 | | 0.734 | -0.918 | 0.176 |
| FT (dev) | 1.604 | 0.421 | -0.046 | | 0.951 | 0.177 | | 0.396 | 0.129 | | 0.738 | -0.305 | 0.785 |
| Two Sites (dev) | 1.070 | 0.295 | 0.274 | | 0.475 | -0.058 | | 0.560 | -0.369 | | 0.264 | -0.259 | 0.649 |
| Three Sites (dev) | 0.041 | 0.974 | 0.008 | | 0.984 | 0.010 | | 0.919 | 0.097 | | 0.796 | -0.757 | 0.162 |
| Four+ Sites (dev) | 1.099 | 0.497 | 0.559 | | 0.236 | 0.028 | | 0.813 | 0.088 | | 0.814 | -0.025 | 0.967 |
| Equivalent Rivals (inv) | -0.996*** | 0.007 | 0.174 | | 0.149 | -0.032 | | 0.253 | 0.096 | | 0.226 | 0.053 | 0.663 |
| Local Area Characteristics | | | | | | | | | | | | | |
| Pop within 30km  (100,000s) (dev) | 1.036 | 0.130 | 0.079 | | 0.764 | -0.103 | | 0.124 | -0.147 | | 0.360 | 0.850*** | 0.001 |
| % pop aged 65+  within 30km (dev) | 2.688 | 0.207 | -0.242 | | 0.721 | 0.084 | | 0.541 | 0.335 | | 0.420 | 0.808 | 0.323 |
| Inc deprivation rank  (1,000s) (inv) | 0.179 | 0.538 | 0.107 | | 0.198 | 0.038* | | 0.096 | 0.029 | | 0.651 | -0.219* | 0.089 |
| Av distance of  nearest GP to  pop (30km) (inv) | -4.810*** | 0.005 | -0.260 | | 0.445 | -0.267** | | 0.035 | 0.591 | | 0.103 | -1.340* | 0.053 |
| MFF (inv) | -17.48 | 0.418 | 7.889 | | 0.348 | 0.844 | | 0.615 | -3.414 | | 0.556 | -15.34 | 0.137 |
| Means of time varying variables (mean) | | | | | | | | | | | | | |
| Bed Categories | | | | | | | | | | | | | |
| Beds 400-549 (mean) | -1.278 | 0.744 | -1.351* | | 0.088 | 0.014 | | 0.936 | -0.456 | | 0.413 | 0.789 | 0.352 |
| Beds 550-699 (mean) | 0.117 | 0.971 | -2.373*** | | 0.003 | -0.062 | | 0.710 | -0.333 | | 0.515 | -0.294 | 0.696 |
| Beds 700-849 (mean) | 0.901 | 0.803 | -3.579*** | | 0.000 | -0.044 | | 0.831 | -0.265 | | 0.666 | -1.185 | 0.148 |
| Beds 850-999 (mean) | -2.439 | 0.482 | -2.807*** | | 0.001 | -0.143 | | 0.470 | -0.510 | | 0.339 | -1.069 | 0.268 |
| Beds 1000-1049 (mean) | -1.855 | 0.607 | -2.377*** | | 0.005 | 0.362 | | 0.136 | -0.270 | | 0.683 | -0.881 | 0.491 |
| Beds 1050+ (mean) | -3.084 | 0.407 | -3.526*** | | 0.000 | -0.181 | | 0.438 | -0.957* | | 0.093 | -1.131 | 0.175 |
| Patient Characteristics | | | | | | | | | | | | | |
| % Age 0-14 (mean) | 0.448 | 0.423 | -0.061 | | 0.663 | -0.048 | | 0.282 | 0.146 | | 0.273 | 0.173 | 0.369 |
| % Age 15-29 (mean) | 0.326 | 0.594 | 0.067 | | 0.679 | 0.042 | | 0.409 | 0.187 | | 0.169 | -0.082 | 0.759 |
| % Age 30-44 (mean) | -0.005 | 0.994 | -0.003 | | 0.993 | -0.053 | | 0.253 | -0.094 | | 0.529 | 0.425 | 0.133 |
| % Age 45-59 (mean) | 0.781 | 0.461 | -0.011 | | 0.964 | -0.012 | | 0.903 | 0.265 | | 0.294 | 0.118 | 0.760 |
| % Age 75-89 (mean) | 1.360 | 0.122 | 0.042 | | 0.860 | 0.027 | | 0.675 | 0.214 | | 0.302 | 0.440 | 0.168 |
| % Age 90+ (mean) | -3.387** | 0.020 | 0.150 | | 0.714 | -0.354*** | | 0.007 | -0.443 | | 0.194 | -0.679 | 0.220 |
| % Male (mean) | -0.174 | 0.676 | -0.025 | | 0.806 | 0.029 | | 0.349 | -0.065 | | 0.447 | -0.021 | 0.865 |
| % Admissions  Emergencies (mean) | 0.149 | 0.199 | -0.012 | | 0.733 | 0.006 | | 0.607 | 0.043 | | 0.154 | 0.035 | 0.436 |
| Trust Characteristics | | | | | | | | | | | | | |
| % Staff doctors (mean) | 0.007 | 0.984 | -0.132 | | 0.274 | 0.017 | | 0.598 | 0.036 | | 0.636 | 0.089 | 0.509 |
| % Staff nurses or  midwives (mean) | 0.529*** | 0.005 | 0.110* | | 0.088 | 0.041** | | 0.013 | -0.028 | | 0.539 | 0.049 | 0.477 |
| % Staff managers (mean) | -1.140* | 0.053 | 0.206 | | 0.265 | -0.031 | | 0.540 | -0.134 | | 0.338 | -0.496** | 0.021 |
| FT (mean) | -0.991 | 0.317 | -0.252 | | 0.293 | -0.117 | | 0.168 | -0.042 | | 0.838 | -0.516 | 0.143 |
| Two Sites (mean) | 0.984 | 0.592 | 0.710* | | 0.084 | 0.066 | | 0.641 | -0.245 | | 0.504 | 0.688 | 0.244 |
| Three Sites (mean) | 4.078** | 0.018 | 1.054*** | | 0.010 | 0.078 | | 0.597 | 0.648 | | 0.111 | 1.684*** | 0.003 |
| Four+ Sites (mean) | 0.957 | 0.620 | 0.557 | | 0.219 | 0.072 | | 0.629 | 0.507 | | 0.204 | 0.276 | 0.675 |
| Local Area Characteristics | | | | | | | | | | | | | |
| Pop within 30km  (100,000s) (mean) | -0.121** | 0.017 | -0.025 | | 0.187 | 0.000 | | 0.938 | 0.007 | | 0.593 | 0.022 | 0.301 |
| % pop aged 65+  within 30km (mean) | 0.137 | 0.707 | -0.069 | | 0.592 | 0.003 | | 0.898 | -0.117 | | 0.119 | 0.187 | 0.182 |
| Observations | 704 | | | 495 | | | 686 | | | 665 | | 678 | |
| R^2 (Overall) | 0.580 | | | 0.234 | | | 0.445 | | | 0.231 | | 0.254 | |
| s.e. | robust | | | robust | | | robust | | | robust | | robust | |
| Mundlak Test | 60.54*** | 0.000 | 43.85** | | 0.021 | 123.8*** | | 0.000 | 56.33*** | | 0.001 | 184.4*** | 0.000 |

Notes: * = p < 0.1, ** = p < 0.05, *** = p < 0.01, robust standard errors are clustered at Trust level. Year and quarter effects are omitted. Mundlak Test: H^o^: all coefficients on means of time varying explanatories are zero.

# Table B2: Patient Complications and Hospital Costs (Within-between specification)

|  | MRSA Rate | | CDif Rate | | RCI | | A&E Waiting times > 4 hours | |
| --- | --- | --- | --- | --- | --- | --- | --- | --- |
|  | b | p | b | p | b | p | b | p |
| Deviations (dev) and time invariant (inv) variables | | | | | | | | |
| Bed Categories | | | | | | | | |
| Beds 400-549 (dev) | 0.164 | 0.585 | 3.375* | 0.074 | 0.621 | 0.519 | -0.495 | 0.300 |
| Beds 550-699 (dev) | -0.057 | 0.882 | 0.661 | 0.835 | 0.100 | 0.948 | 0.101 | 0.879 |
| Beds 700-849 (dev) | 0.037 | 0.927 | 1.656 | 0.636 | 1.564 | 0.366 | 0.179 | 0.823 |
| Beds 850-999 (dev) | 0.166 | 0.703 | 0.319 | 0.927 | 1.689 | 0.363 | 0.403 | 0.647 |
| Beds 1000-1049 (dev) | 0.416 | 0.411 | 1.310 | 0.720 | 0.658 | 0.747 | 0.106 | 0.904 |
| Beds 1050+ (dev) | 0.029 | 0.954 | 1.178 | 0.770 | 0.321 | 0.884 | 0.583 | 0.526 |
| Patient Characteristics | | | | | | | | |
| % Age 0-14 (dev) | -0.004 | 0.972 | 0.620 | 0.409 | 0.125 | 0.735 | 0.307 | 0.361 |
| % Age 15-29 (dev) | -0.159 | 0.262 | 0.262 | 0.794 | 0.454 | 0.457 | -0.253 | 0.447 |
| % Age 30-44 (dev) | 0.196 | 0.165 | 1.472 | 0.301 | -0.413 | 0.507 | 0.619 | 0.119 |
| % Age 45-59 (dev) | -0.135 | 0.382 | 0.155 | 0.897 | 0.356 | 0.571 | -0.804 | 0.016 |
| % Age 75-89 (dev) | -0.094 | 0.458 | 0.249 | 0.798 | -0.306 | 0.566 | 0.271 | 0.436 |
| % Age 90+ (dev) | 0.059 | 0.784 | 1.201 | 0.551 | 0.032 | 0.975 | -0.944 | 0.275 |
| % Male (dev) | 0.057 | 0.473 | 0.353 | 0.619 | 0.354 | 0.371 | 0.324 | 0.238 |
| % Admissions  Emergencies (dev) | 0.013 | 0.611 | -0.128 | 0.499 | 0.041 | 0.715 | -0.047 | 0.456 |
| Trust Characteristics | | | | | | | | |
| % Staff doctors (dev) | 0.105* | 0.063 | 0.489 | 0.111 | -0.057 | 0.710 | -0.156 | 0.193 |
| % Staff nurses or  midwives (dev) | 0.021 | 0.654 | 0.777* | 0.076 | 0.295 | 0.133 | 0.003 | 0.986 |
| % Staff managers (dev) | -0.011 | 0.931 | 0.972 | 0.404 | -1.520** | 0.013 | -0.733** | 0.037 |
| Teaching (inv) | 0.011 | 0.955 | 2.858* | 0.068 | 0.133 | 0.924 | 0.142 | 0.780 |
| FT (dev) | 0.569** | 0.013 | 2.749 | 0.306 | -4.007** | 0.027 | 0.344 | 0.442 |
| Two Sites (dev) | 0.150 | 0.424 | -1.140 | 0.411 | -0.693 | 0.538 | -0.193 | 0.866 |
| Three Sites (dev) | 0.153 | 0.479 | -0.674 | 0.675 | 0.157 | 0.887 | -0.256 | 0.803 |
| Four+ Sites (dev) | -0.285 | 0.307 | -1.163 | 0.501 | 0.107 | 0.927 | 0.535 | 0.583 |
| Equivalent Rivals (inv) | 0.025 | 0.390 | -0.139 | 0.659 | 0.150 | 0.600 | -0.046 | 0.638 |
| Local Area Characteristics | | | | | | | | |
| Pop within 30km  (100,000s) (dev) | -0.196* | 0.052 | 1.764 | 0.103 | 0.585 | 0.186 | 0.021 | 0.948 |
| % pop aged 65+  within 30km (dev) | 0.136 | 0.628 | 7.002*** | 0.009 | 2.901** | 0.022 | 1.337 | 0.217 |
| Inc deprivation  rank (1,000s) (inv) | -0.018 | 0.539 | -0.197 | 0.463 | 0.139 | 0.598 | -0.078 | 0.341 |
| Av distance of  nearest GP to  pop (30km) (inv) | -0.017 | 0.892 | -0.568 | 0.680 | 1.611 | 0.277 | 0.511 | 0.296 |
| MFF (inv) | -2.877 | 0.301 | -46.49** | 0.033 | 11.96 | 0.465 | -10.34 | 0.138 |
| Means of time varying variables (mean) | | | | | | | | |
| Bed Categories | | | | | | | | |
| Beds 400-549 (mean) | 0.068 | 0.739 | 0.946 | 0.737 | -2.951 | 0.108 | 0.154 | 0.829 |
| Beds 550-699 (mean) | -0.204 | 0.287 | 0.085 | 0.969 | -5.659*** | 0.001 | 1.115* | 0.074 |
| Beds 700-849 (mean) | -0.217 | 0.290 | 2.187 | 0.385 | -4.618** | 0.022 | 1.090 | 0.162 |
| Beds 850-999 (mean) | -0.145 | 0.568 | 2.000 | 0.433 | -3.614* | 0.084 | 0.930 | 0.209 |
| Beds 1000-1049 (mean) | 0.075 | 0.771 | 2.433 | 0.349 | -3.491 | 0.126 | 2.471** | 0.014 |
| Beds 1050+ (mean) | -0.159 | 0.499 | 0.833 | 0.727 | -2.679 | 0.165 | 0.955 | 0.233 |
| Patient Characteristics | | | | | | | | |
| % Age 0-14 (mean) | 0.058 | 0.271 | 0.382 | 0.359 | -0.058 | 0.900 | -0.061 | 0.674 |
| % Age 15-29 (mean) | 0.082* | 0.099 | -0.552 | 0.249 | 0.107 | 0.831 | 0.097 | 0.583 |
| % Age 30-44 (mean) | 0.073 | 0.208 | 0.313 | 0.583 | -0.295 | 0.570 | -0.051 | 0.734 |
| % Age 45-59 (mean) | 0.108 | 0.288 | 0.753 | 0.359 | 1.029 | 0.237 | -0.169 | 0.527 |
| % Age 75-89 (mean) | 0.053 | 0.497 | 0.390 | 0.549 | -0.610 | 0.399 | -0.130 | 0.641 |
| % Age 90+ (mean) | 0.101 | 0.494 | -1.580 | 0.263 | 0.216 | 0.861 | 0.388 | 0.311 |
| % Male (mean) | 0.034 | 0.273 | 0.025 | 0.934 | 0.230 | 0.475 | 0.024 | 0.797 |
| % Admissions  Emergencies (mean) | 0.018* | 0.076 | 0.016 | 0.876 | -0.071 | 0.452 | 0.032 | 0.383 |
| Trust Characteristics | | | | | | | | |
| % Staff doctors (mean) | 0.067*** | 0.008 | -0.009 | 0.972 | -0.734*** | 0.001 | 0.183* | 0.062 |
| % Staff nurses or  midwives (mean) | 0.021 | 0.256 | -0.110 | 0.551 | 0.080 | 0.562 | -0.003 | 0.952 |
| % Staff managers (mean) | -0.002 | 0.980 | 0.408 | 0.429 | 0.045 | 0.926 | 0.310 | 0.065 |
| FT (mean) | -0.070 | 0.392 | -0.442 | 0.608 | -1.578* | 0.065 | -0.927*** | 0.002 |
| Two Sites (mean) | 0.101 | 0.523 | -2.537* | 0.082 | 2.043 | 0.105 | 1.155** | 0.014 |
| Three Sites (mean) | 0.245 | 0.126 | -2.880** | 0.049 | 1.924 | 0.144 | 0.558 | 0.288 |
| Four+ Sites (mean) | 0.400** | 0.017 | -0.622 | 0.693 | -0.365 | 0.779 | 0.033 | 0.952 |
| Local Area Characteristics | | | | | | | | |
| Pop within 30km  (100,000s) (mean) | 0.007 | 0.238 | 0.104** | 0.032 | 0.024 | 0.573 | -0.002 | 0.893 |
| % pop aged 65+  within 30km (mean) | 0.002 | 0.953 | 0.360 | 0.174 | 0.322 | 0.229 | -0.174* | 0.074 |
| Constant | -4.172 | 0.369 | 52.77 | 0.186 | 78.20 | 0.100 | 0.998 | 0.953 |
| Observations | 702 | | 704 | | 704 | | 2235 | |
| R^2 (Overall) | 0.315 | | 0.423 | | 0.354 | | 0.413 | |
| s.e. | robust | | robust | | robust | | Robust | |
| Mundlak Test | 75.96*** | 0.000 | 46.90** | 0.010 | 159.4*** | 0.000 | 1626*** | 0.000 |

Notes: * = p < 0.1, ** = p < 0.05, *** = p < 0.01, robust standard errors are clustered at Trust level. Mundlak test: H^o^: all coefficients on means of time varying explanatories are zero. Year and quarter effects are omitted.

# Table B3: Patient experience (Within-between specification)

|  | Cleanliness Score | | Involvement Score | | Dignity Score | | | FFT  Recommendation | |
| --- | --- | --- | --- | --- | --- | --- | --- | --- | --- |
|  | b | p | b | p | b | | p | b | p |
| Deviations (dev) and time invariant (inv) variables | | | | | | | | | |
| Bed Categories | | | | | | | | | |
| Beds 400-549 (dev) | -0.582 | 0.288 | -0.456 | 0.524 | 0.076 | | 0.863 | 1.808** | 0.013 |
| Beds 550-699 (dev) | -0.874 | 0.287 | -1.277 | 0.166 | 0.237 | | 0.716 | 2.380*** | 0.003 |
| Beds 700-849 (dev) | -0.069 | 0.945 | -1.479 | 0.174 | 0.433 | | 0.590 | 1.460* | 0.096 |
| Beds 850-999 (dev) | -0.709 | 0.495 | 0.086 | 0.947 | 0.436 | | 0.623 | -0.185 | 0.925 |
| Beds 1000-1049 (dev) | -0.644 | 0.554 | -0.953 | 0.460 | -0.066 | | 0.943 | 0.176 | 0.928 |
| Beds 1050+ (dev) | -0.185 | 0.870 | -0.436 | 0.748 | 0.503 | | 0.627 | 2.904** | 0.017 |
| Patient Characteristics | | | | | | | | | |
| % Age 0-14 (dev) | -0.260 | 0.134 | -0.271 | 0.274 | 0.076 | | 0.697 | § |  |
| % Age 15-29 (dev) | 0.030 | 0.910 | -0.063 | 0.863 | 0.323 | | 0.270 | § |  |
| % Age 30-44 (dev) | -0.574** | 0.025 | -0.485 | 0.211 | -0.326 | | 0.272 | § |  |
| % Age 45-59 (dev) | 0.333 | 0.231 | 0.153 | 0.711 | 0.384 | | 0.255 | § |  |
| % Age 75-89 (dev) | -0.385* | 0.093 | -0.398 | 0.222 | -0.048 | | 0.855 | § |  |
| % Age 90+ (dev) | -0.164 | 0.755 | -0.178 | 0.779 | -0.019 | | 0.972 | § |  |
| % Male (dev) | -0.082 | 0.631 | -0.008 | 0.973 | 0.166 | | 0.354 | § |  |
| % Admissions  Emergencies (dev) | 0.034 | 0.556 | -0.063 | 0.390 | -0.025 | | 0.676 | § |  |
| Trust Characteristics | | | | | | | | | |
| % Staff doctors (dev) | -0.254** | 0.016 | -0.200 | 0.219 | -0.155 | | 0.166 | § |  |
| % Staff nurses or  midwives (dev) | -0.002 | 0.977 | -0.059 | 0.635 | -0.006 | | 0.953 | § |  |
| % Staff managers (dev) | -0.343 | 0.189 | 0.678 | 0.131 | 0.519* | | 0.097 | § |  |
| Teaching (inv) | 0.674 | 0.356 | 1.259** | 0.039 | 1.332*** | | 0.008 | -0.739 | 0.405 |
| FT (dev) | 0.627 | 0.138 | 0.567 | 0.427 | 0.640 | | 0.190 | § |  |
| Two Sites (dev) | -0.266 | 0.550 | -0.471 | 0.372 | -0.050 | | 0.911 | § |  |
| Three Sites (dev) | -0.746 | 0.144 | -0.526 | 0.369 | -0.201 | | 0.672 | § |  |
| Four+ Sites (dev) | -0.160 | 0.773 | 0.257 | 0.702 | 0.625 | | 0.253 | § |  |
| Equivalent Rivals (inv) | 0.203* | 0.097 | 0.179 | 0.125 | 0.189** | | 0.038 | 0.096 | 0.596 |
| Local Area Characteristics | | | | | | | | | |
| Pop within 30km  (100,000s) (dev) | 0.224 | 0.322 | 0.363 | 0.252 | 0.292 | | 0.187 | § |  |
| % pop aged 65+ within  30km (dev) | 0.972 | 0.135 | 0.907 | 0.262 | 0.417 | | 0.531 | § |  |
| Inc deprivation rank  (1,000s) (inv) | 0.071 | 0.519 | -0.096 | 0.302 | -0.099 | | 0.169 | -0.516*** | 0.000 |
| Av distance of  nearest GP to  pop (30km) (inv) | -0.808 | 0.161 | 0.739 | 0.202 | -0.337 | | 0.469 | -0.582 | 0.567 |
| MFF (inv) | 0.160 | 0.986 | -3.021 | 0.700 | 1.784 | | 0.766 | -27.62** | 0.049 |
| Means of time varying variables (mean) | | | | | | | | | |
| Bed Categories | | | | | | | | | |
| Beds 400-549 (mean) | 0.918 | 0.267 | -0.331 | 0.726 | 0.012 | | 0.985 | -0.936 | 0.379 |
| Beds 550-699 (mean) | 0.541 | 0.488 | -0.830 | 0.371 | 0.219 | | 0.724 | 0.257 | 0.808 |
| Beds 700-849 (mean) | 1.991** | 0.047 | -0.184 | 0.857 | 0.832 | | 0.292 | 1.543 | 0.265 |
| Beds 850-999 (mean) | 0.699 | 0.427 | 0.222 | 0.820 | 0.553 | | 0.394 | -0.132 | 0.935 |
| Beds 1000-1049 (mean) | 1.472 | 0.204 | 0.274 | 0.797 | 0.822 | | 0.257 | 0.542 | 0.741 |
| Beds 1050+ (mean) | 1.177 | 0.203 | 0.281 | 0.785 | 0.545 | | 0.477 | 0.204 | 0.884 |
| Patient Characteristics | | | | | | | | | |
| % Age 0-14 (mean) | -0.243 | 0.158 | 0.025 | 0.887 | -0.248* | | 0.054 | -0.083 | 0.703 |
| % Age 15-29 (mean) | -0.249 | 0.188 | -0.221 | 0.201 | -0.451*** | | 0.001 | 0.036 | 0.900 |
| % Age 30-44 (mean) | -0.229 | 0.308 | 0.100 | 0.600 | -0.023 | | 0.888 | -0.464 | 0.118 |
| % Age 45-59 (mean) | -0.045 | 0.889 | 0.240 | 0.483 | -0.255 | | 0.323 | 0.110 | 0.790 |
| % Age 75-89 (mean) | -0.476 | 0.104 | -0.293 | 0.258 | -0.427** | | 0.031 | -0.170 | 0.713 |
| % Age 90+ (mean) | 0.396 | 0.481 | 1.038** | 0.048 | 0.152 | | 0.719 | 0.235 | 0.702 |
| % Male (mean) | -0.053 | 0.686 | -0.058 | 0.639 | -0.064 | | 0.469 | 0.055 | 0.712 |
| % Admissions  Emergencies (mean) | -0.059 | 0.220 | -0.093** | 0.026 | -0.030 | | 0.332 | -0.185** | 0.024 |
| Trust Characteristics | | | | | | | | | |
| % Staff doctors (mean) | -0.049 | 0.723 | -0.275** | 0.023 | -0.211** | | 0.032 | -0.099 | 0.285 |
| % Staff nurses or  midwives (mean) | -0.011 | 0.866 | -0.100 | 0.128 | 0.019 | | 0.687 | 0.153 | 0.117 |
| % Staff managers (mean) | -0.374 | 0.143 | 0.201 | 0.319 | 0.134 | | 0.379 | -0.107 | 0.790 |
| FT (mean) | 0.342 | 0.394 | 1.138*** | 0.004 | 0.684** | | 0.013 | 0.290 | 0.581 |
| Two Sites (mean) | -1.117* | 0.059 | -0.400 | 0.487 | -0.260 | | 0.539 | -0.025 | 0.974 |
| Three Sites (mean) | -1.183* | 0.056 | -0.781 | 0.168 | -1.166*** | | 0.007 | -1.872 | 0.121 |
| Four+ Sites (mean) | -1.049 | 0.104 | -0.648 | 0.264 | -0.713 | | 0.130 | -0.678 | 0.471 |
| Local Area Characteristics | | | | | | | | | |
| Pop within 30km  (100,000s) (mean) | -0.039* | 0.086 | -0.009 | 0.602 | -0.027** | | 0.032 | 0.080** | 0.011 |
| % pop aged 65+  within 30km (mean) | 0.180 | 0.157 | 0.244* | 0.080 | 0.196 | | 0.105 | 0.058 | 0.794 |
| Constant | 111.4*** | 0.000 | 82.50*** | 0.000 | 111.7*** | | 0.000 | 132.6*** | 0.000 |
| Observations | 568 | |  | | 568 | | | 532 | |
| R^2 (Overall) | 0.393 | | 0.486 | | 0.436 | | | 0.228 | |
| s.e. | robust | | robust | | robust | | | Robust | |
| Mundlak Test | 64.07*** | 0.000 | 120.4*** | 0.000 | | 142.3*** | 0.000 | 63.05*** | 0.000 |

Notes: * = p < 0.1, ** = p < 0.05, *** = p < 0.01, robust standard errors are clustered at Trust level. Mundlak test: H^o^: all coefficients on means of time varying explanatories are zero. Year and quarter effects are omitted. dev: deviation of time varying variable from its mean. inv: variable is constant over time.

# Appendix C: Analysis with single-site Trusts (Model 2)

|  | *Overall mortality*  *SHMI* | | *AMI (heart attack)*  *Mortality Rate* | | *Non-Elective*  *Mortality Rate* | | *Hip Fracture*  *Mortality Rate* | | *Stroke*  *Mortality Rate* | |
| --- | --- | --- | --- | --- | --- | --- | --- | --- | --- | --- |
|  | coeff | p-value | coeff | p-value | coeff | p-value | coeff | p-value | coeff | p-value |
| Beds 400-549 | 1.653 | 0.513 | -0.104 | 0.870 | 0.278** | 0.037 | 0.346 | 0.364 | 1.193** | 0.041 |
| Beds 550-699 | 3.036 | 0.250 | -1.25* | 0.070 | 0.255* | 0.072 | -0.255 | 0.509 | 0.725 | 0.309 |
| Beds 700+ | 3.017 | 0.286 | -1.46* | 0.078 | 0.284 | 0.240 | -0.540 | 0.255 | 0.018 | 0.984 |
| Observations | 240 |  | 130 |  | 231 |  | 222 |  | 225 |  |
| Breusch-Pagan Test | 109.0*** | 0.000 | 1.050 | 0.153 | 7.762*** | 0.003 | 1.092 | 0.148 | 0.050 | 0.412 |
| Mundlak Test | 29.41 | 0.105 | 79.39*** | 0.000 | 341.2*** | 0.000 | 814.6*** | 0.000 | 597.3*** | 0.000 |
| R^2^ (Overall) | 0.473 |  | 0.369 |  | 0.436 |  | 0.342 |  | 0.223 |  |
| s.e. | robust |  | robust |  | robust |  | robust |  | robust |  |
|  | *MRSA infections rate* | | *C-Difficile*  *infections rate* | | *Reference Cost Index* | | *A&E waiting times > 4 hours* | |  | |
| Beds 400-549 | 0.143 | 0.522 | 2.490 | 0.198 | -0.936 | 0.325 | -0.442 | 0.224 |  |  |
| Beds 550-699 | -0.244 | 0.254 | -2.827 | 0.178 | -3.524*** | 0.002 | -0.016 | 0.971 |  |  |
| Beds 700+ | -0.255 | 0.342 | 3.952* | 0.097 | -0.344 | 0.854 | 0.427 | 0.633 |  |  |
| Observations | 239 |  | 240 |  | 240 |  | 718 |  |  |  |
| Breusch-Pagan Test | 0.000 | 0.496 | 19.08*** | 0.000 | 45.58*** | 0.000 | 288.6*** | 0.000 |  |  |
| Mundlak Test | 107.0*** | 0.000 | 30.12 | 0.115 | 161.6*** | 0.000 | 39.03** | 0.014 |  |  |
| R^2^ (Overall) | 0.287 |  | 0.373 |  | 0.356 |  | 0.169 |  |  |  |
| s.e. | robust |  | robust |  | robust |  | Robust |  |  |  |
|  | *Cleanliness Score* | | *Involvement Score* | | *Dignity Score* | | *FFT*  *Recommendation* | |  | |
| Beds 400-549 | -0.874** | 0.010 | -0.322 | 0.623 | -0.261 | 0.568 | 0.041 | 0.971 |  |  |
| Beds 550-699 | -0.780 | 0.302 | -0.917 | 0.291 | 0.255 | 0.639 | 0.818 | 0.446 |  |  |
| Beds 700+ | 0.307 | 0.766 | -0.994 | 0.339 | -0.134 | 0.860 | 0.996 | 0.308 |  |  |
| Observations | 203 |  | 203 |  | 203 |  | 146 |  |  |  |
| Breusch-Pagan Test | 103.6*** | 0.000 | 29.21*** | 0.000 | 34.22*** | 0.000 | 23.09*** | 0.000 |  |  |
| Mundlak Test | 34.94** | 0.014 | 62.20*** | 0.000 | 43.62*** | 0.001 | 60.48*** | 0.000 |  |  |
| R^2^ (Overall) | 0.456 |  | 0.532 |  | 0.500 |  | 0.427 |  |  |  |
| s.e. | robust |  | robust |  | robust |  | robust |  |  |  |

Notes: control variables are not reported.

# Appendix D: Friends and Family Test Score, 2013-14

|  | *Model 1* | | *Model 2* | |
| --- | --- | --- | --- | --- |
|  | coeff | p-value | coeff | p-value |
| Beds 400-549 | -3.044* | 0.076 | -2.608 | 0.242 |
| Beds 550-699 | -2.098 | 0.200 | -2.471 | 0.254 |
| Beds 700-849 | 0.168 | 0.915 | -1.850 | 0.441 |
| Beds 850-999 | -1.783 | 0.263 | -0.690 | 0.778 |
| Beds 1000-1049 | -1.999 | 0.270 | -1.674 | 0.529 |
| Beds 1150+ | 0.084 | 0.959 | 0.832 | 0.759 |
| Beds 700+ |  |  |  |  |
| Constant | 183.7*** | 0.000 | 123.1** | 0.047 |
| Observations | 560 |  | 560 |  |
| Breusch-Pagan Test |  |  | 470.0*** | 0.000 |
| R^2^ | 0.182 |  | 0.267 |  |

Notes: * = p < 0.1, ** = p < 0.05, *** = p < 0.01, robust standard errors are clustered at Trust level. Control variables not reported.

# Appendix E: Comparison of marginal effects from OLS and Fractional logit

As a robustness check, we also estimate a fractional logit model to allow for the dependent variables being in most cases percentages or proportions and thus bounded. We use the Stata command fracreg. Compared to other regression methods to model rates or fractional outcomes such as beta regressions, the fractional logit specification can allow for observations which take on the minimum or maximum bound.

We compare the marginal effects of the hospital size categories from the OLS and fractional logit regressions with the full set of explanatories. We do not include hospital random effects in either model because fracreg does not allow for them. We plot the marginal effects and their 95% confidence intervals for all seven size categories. The results are very similar, which reassures about the robustness of our empirical strategy.


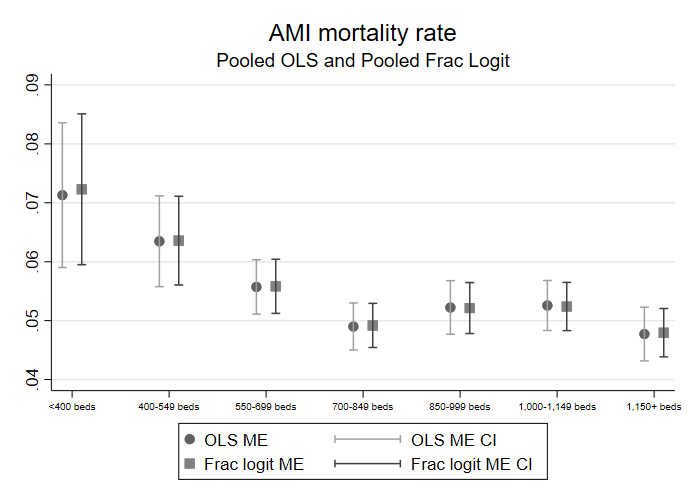

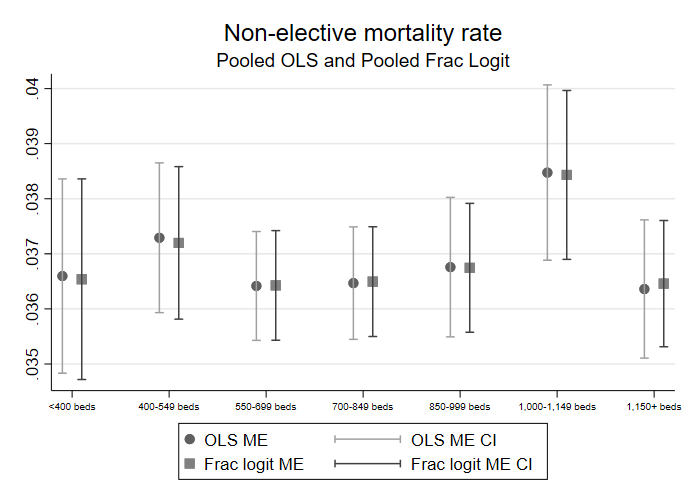

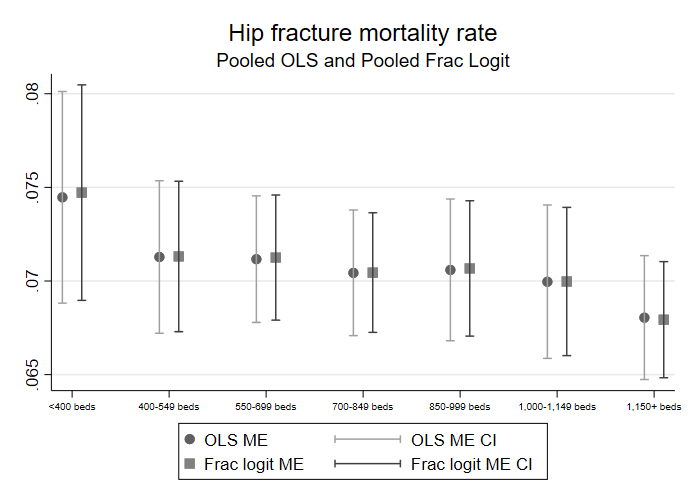

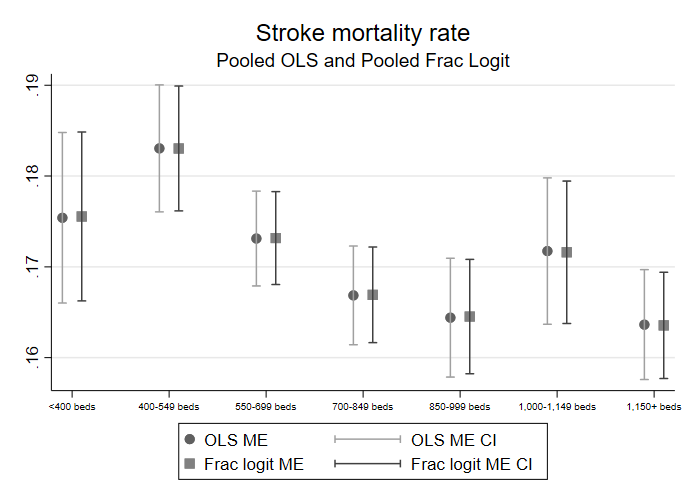

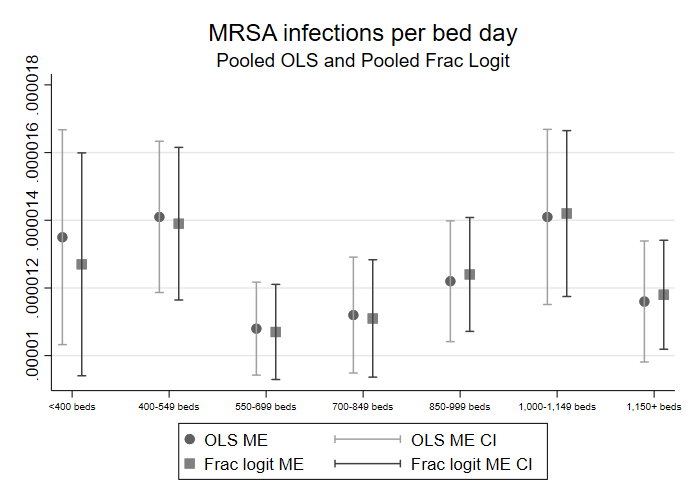

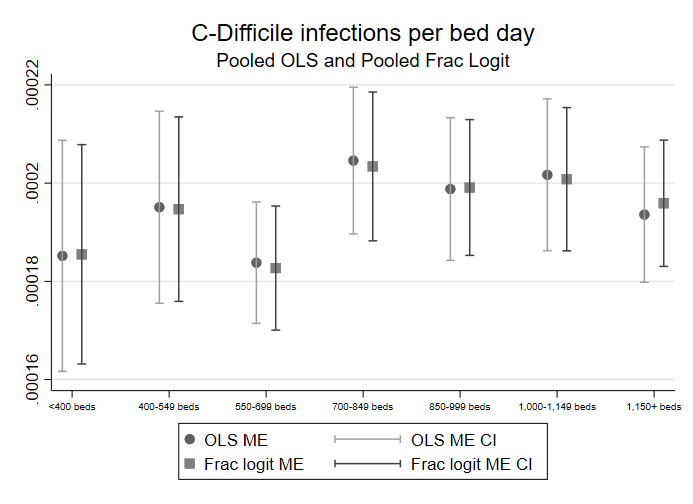

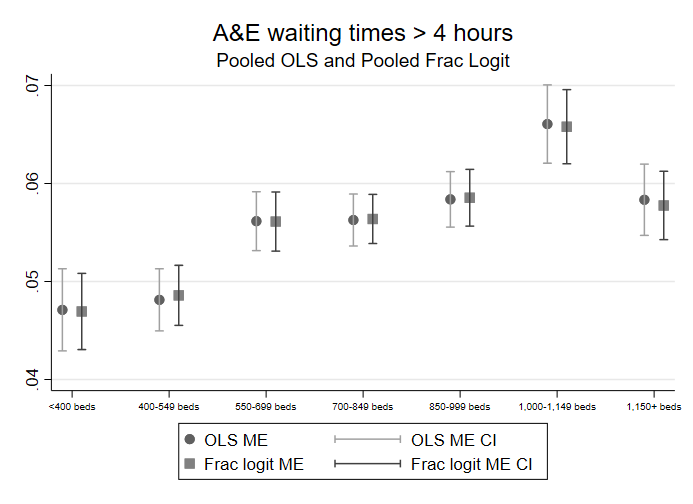

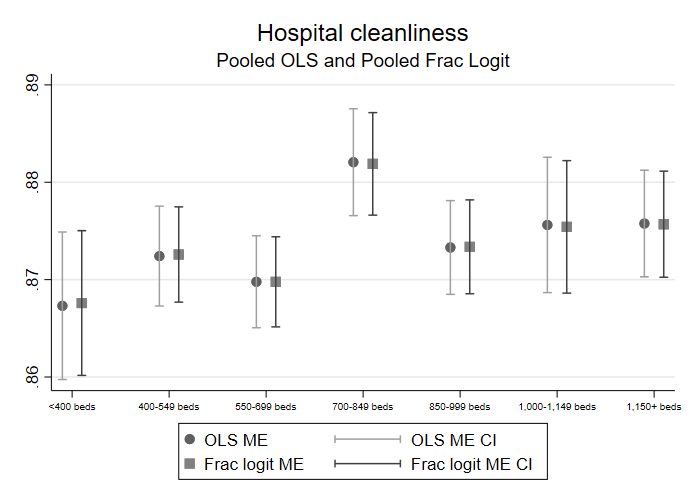

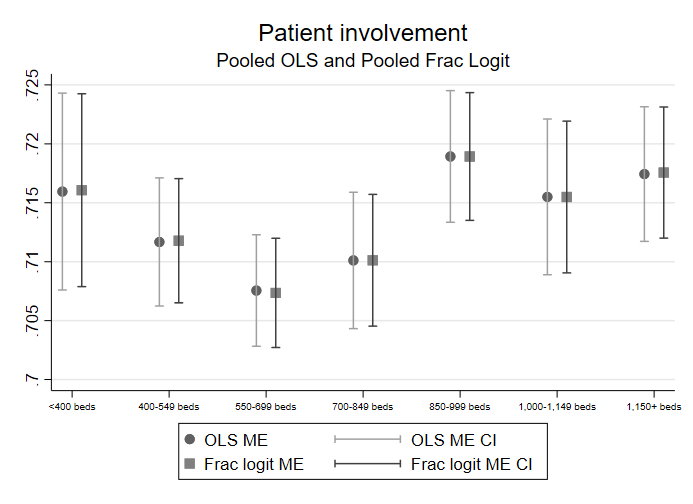

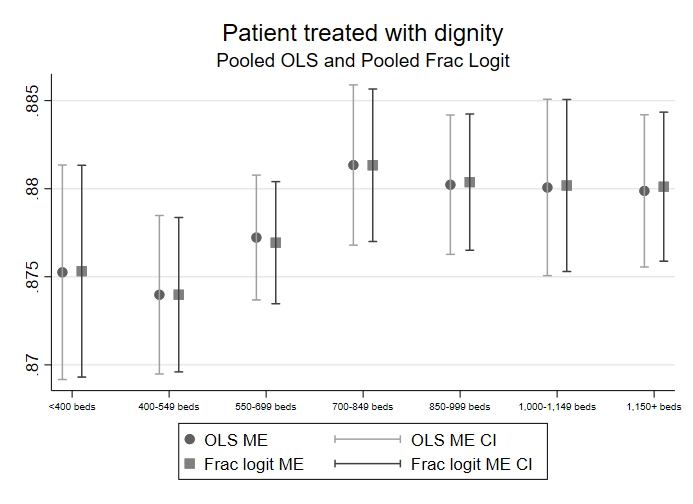


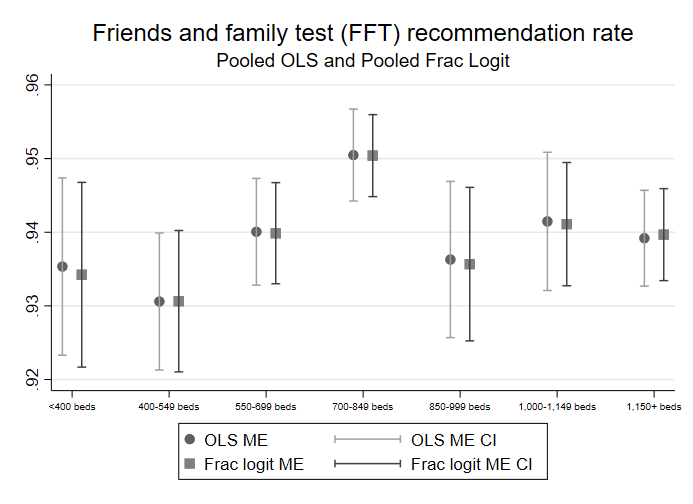


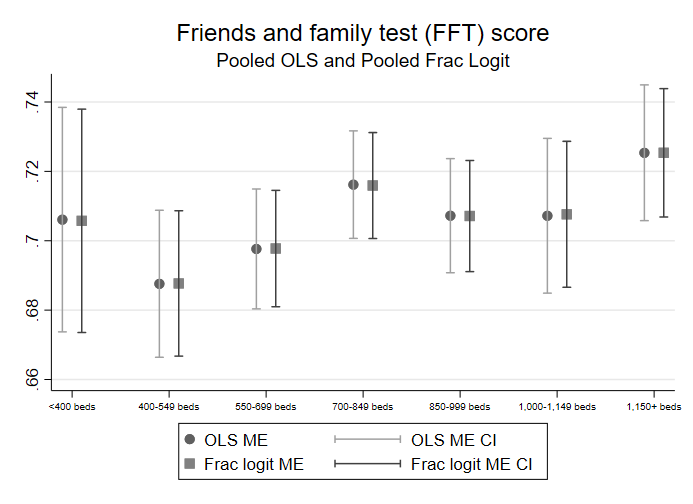

Supplement: Multimedia component 1 [file mmc1.docx]
